# Selection and Validation of Reference Genes for Quantitative RT-PCR Analysis in *Corylus heterophylla* Fisch. *× Corylus avellana* L.

**DOI:** 10.3390/plants10010159

**Published:** 2021-01-15

**Authors:** Sihao Hou, Tiantian Zhao, Dan Yang, Qing Li, Lisong Liang, Guixi Wang, Qinghua Ma

**Affiliations:** 1 Key Laboratory of Tree Breeding and Cultivation of the State Forestry and Grassland Administration, Research Institute of Forestry, Chinese Academy of Forestry, Beijing 100091, China; HSH@caf.ac.cn (S.H.); ztt@caf.ac.cn (T.Z.); YD@caf.ac.cn (D.Y.); LQ@caf.ac.cn (Q.L.); lianglisong@caf.ac.cn (L.L.); WGX@caf.ac.cn (G.W.); 2 Hazelnut Engineering and Technical Research Center of the State Forestry and Grassland Administration, Beijing 100091, China; 3 National Hazelnut Industry Innovation Alliance of the State Forestry and Grassland Administration, Beijing 100091, China

**Keywords:** Ping’ou hybrid hazelnut (*C. heterophylla* Fisch. × *C. avellana* L.), real-time quantitative PCR, reference gene, stability of gene expression, self-incompatibility

## Abstract

(1) Background: the species of *Corylus* have sporophytic type of self-incompatibility. Several genes related to recognition reaction between pollen and stigma have been identified in hazelnuts. To better understand the self-incompatibility (SI) response, we screened the suitable reference genes by using quantitative real-time reverse transcription PCR (qRT-PCR) analysis in hazelnut for the first time. (2) Methods: the major cultivar “*Dawei*” was used as material. A total of 12 candidate genes were identified and their expression profiles were compared among different tissues and in response to various treatments (different times after self- and cross-pollination) by RT-qPCR. The expression stability of these 12 candidate reference genes was evaluated using geNorm, NormFinder, BestKeeper, Delta Ct, and RefFinder programs. (3) Results: the comprehensive ranking of RefFinder indicated that *ChaActin*, *VvActin,* *ChaUBQ14,* and *ChaEF1-α* were the most suitable reference genes. According to the stability analysis of 12 candidate reference genes for each sample group based on four software packages, *ChaActin* and *ChaEF1-α* were most stable in different times after self-pollination and 4 h after self- and cross-pollination, respectively. To further validate the suitability of the reference genes identified in this study, *CavPrx*, which the expression profiles in *Corylus* have been reported, was quantified by using *ChaActin* and *ChaEF1-α* as reference genes. (4) Conclusions: our study of reference genes selection in hazelnut shows that the two reference genes, *ChaActin* and *ChaEF1-α*, are suitable for the evaluation of gene expression, and can be used for the analysis of pollen-pistil interaction in *Corylus*. The results supply a reliable foundation for accurate gene quantifications in *Corylus* species, which will facilitate the studies related to the reproductive biology in *Corylus.*

## 1. Introduction

Hazelnut (*Corylus*), a member of the birch family (Betulaceae) in the order Fagales, is one of the most important nut crops and a woody oil plant, with high economic and nutritional value. To date, around 25 species of *Corylus* have been described by taxonomists, among which 13 are widely-recognized [1,2]. The various species of hazelnut are primarily distributed across the temperate zones of the Northern Hemisphere [3], with Asia, Europe, and North America. Among them, only some cultivars of European hazelnut (*C. avellana*) have been commercialized [4,5]. Approximately eight species and two varieties of *Corylus* are native to China, and are widely-distributed across 24 provinces. The main commercial cultivars are several types of Ping’ou hybrid hazelnuts, obtained by artificial interspecific hybridization of the Ping hazelnut (*C. heterophylla*) with European hazelnut [6,7].

According to world *Corylus* resources, world hazelnut species exhibit abundant characteristics, such as tree features, nut characteristics, stress resistance, and self-incompatibility, deserving more investigation and application. Hazelnut researchers are currently focusing on breeding for Eastern Filbert Blight resistance in European hazelnut [8,9], S-locus gene identification and distribution among cultivars and species [10,11], and cold-resistance breeding of interspecies hybrids with some wild species as the female parent [12,13]. As these studies progress, the use of molecular biotechnology is of great significance for the identification of trait-specific genes and molecular-assisted breeding in hazelnut.

Quantitative real-time polymerase chain reaction (RT-qPCR) is among the most common methods used for gene expression and transcriptome analysis. It refers to an improved PCR technique, which uses fluorescent probes and fluorescent dyes in the reaction system, and is characterized by high sensitivity and specificity, good reproducibility, a wide dynamic quantification range, and high-throughput capacity for a limited number of target genes [14]. RT-qPCR is a powerful tool for RNA analysis, conducted using cDNA template, which is generated from RNA. The initial RNA quantity, purity, and the efficiencies of reverse transcription and amplification, all affect the accuracy of gene expression analysis by RT-qPCR [15,16]. Reference genes are commonly used for standardization, to minimize experimental error [16]. Increasing studies have shown that an ideal reference gene is expressed stably, or relatively stable, regardless of tissue or cell type, and in the presence of various test environments and influencing factors [17,18,19]. Traditional reference genes, such as actin (*ACT*), glyceraldehyde-3-phosphate dehydrogenase (*GAPDH*), ubiquitin (*UBQ*), and *18S rRNA* have been tested in previous investigations. However, variations in the expression levels of reference genes have been reported in different tissues and/or in response to experimental treatments. Therefore, it is a challenge for RT-qPCR to identify a set of reference genes whose mRNA expression levels do not change significantly across tissues, independently of the experimental context [20]. In most cases, validation of reference genes is determined using statistical approaches, such as geNorm [21], NormFinder [22], BestKeeper [23], and Delta Ct [24].

Hazelnuts are monoecious, wind-pollinated, and self-incompatible. Reproduction is restricted by a sporophytic self-incompatibility system, which is controlled by a single locus with various S-alleles determining compatibility [25]. Self-incompatibility is considered to be an evolutionary advantage, but it is also a limiting factor for obtaining a commercial yield in hazelnut, since it restricts the choice of some cultivars that can be used in the same plantation. Consequently, knowledge of specific S-alleles associated with each cultivar, as well as the type of genetic control of incompatibility would greatly facilitate the choice of successful parental combinations. Several self-incompatibility-related genes to date have been identified in hazelnut [26,27,28]. However, there are no reports of systematic identification of reference genes suitable for use in the evaluation of self-incompatibility-related genes in RT-qPCR expression studies. Thus, the identification of reliable reference genes for use in RT-qPCR will benefit further studies using this mature technology by reducing costs. Furthermore, the using of appropriate reference genes facilitates more accurate calculation of gene expression levels and provides a useful resource for future research. In this study, the expression stability of 12 candidate reference genes, selected based on transcriptome data from the Ping’ou hybrid hazelnut and previous studies, was analyzed by RT-qPCR in different plant materials, using four statistical algorithms for reference gene detection. The objective of this study was to validate suitable reference genes, which may help to facilitate subsequent studies of the expression profiles of self-incompatibility related genes.

## 2. Results

### 2.1. Identification of Candidate Reference Genes and Primer Specificity and Efficiency

According to the filtering criteria (False Discovery Rate (FDR) < 0.01 and Log2 ratio between −1 and 1) mentioned in the Materials and Methods, three candidate reference genes were selected from the transcriptome database, based on expression stability. Homologous sequences of seven traditional reference genes were also selected as candidates; however, the expression levels of sequences homologous to 18SrRNA and β-tubulin (TUB) showed distinct differences among treatments, and these two genes were considered unsuitable as reference genes. The remaining five homologous sequences were selected as candidate reference genes. Therefore, there were eight candidate reference genes (Table 1). Four reported traditional reference genes (*ChActin* for *C. heterophylla* Fisch. [12], *VvUBQ*, *VvActin* for *Corylus avellana* L. [28] and *Cha18S rRNA* for *C. heterophylla* Fisch. *× Corylus avellana* L. [29]) in hazelnuts were also selected.

Twelve pairs of primers were generated by software design and literature review (Table 2), and they produced standard curves demonstrating good amplification efficiency (E ranged from 86.3% to 121.6%) and there was a linear relationship (R2 > 0.980) for all tested reference genes, confirming the suitability of the primer pairs for RT-qPCR-based quantification.

**Figure 1:**
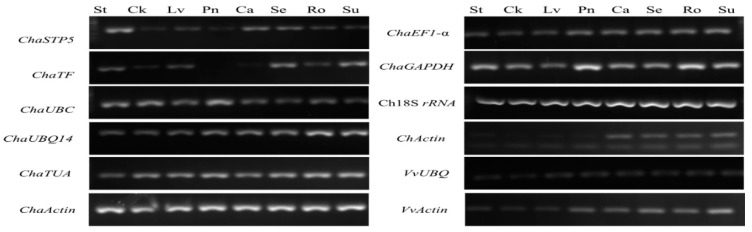
Agarose electrophoresis of 12 candidate reference gene RT-PCR products amplified from different samples. St, blooming styles; Ck, catkins before elongation; Lv, young leaves; Pn, pollen; Ca, cambium of annual branch; Se, green stem; Ro, root tip; Su, sucker.

**Figure 2:**
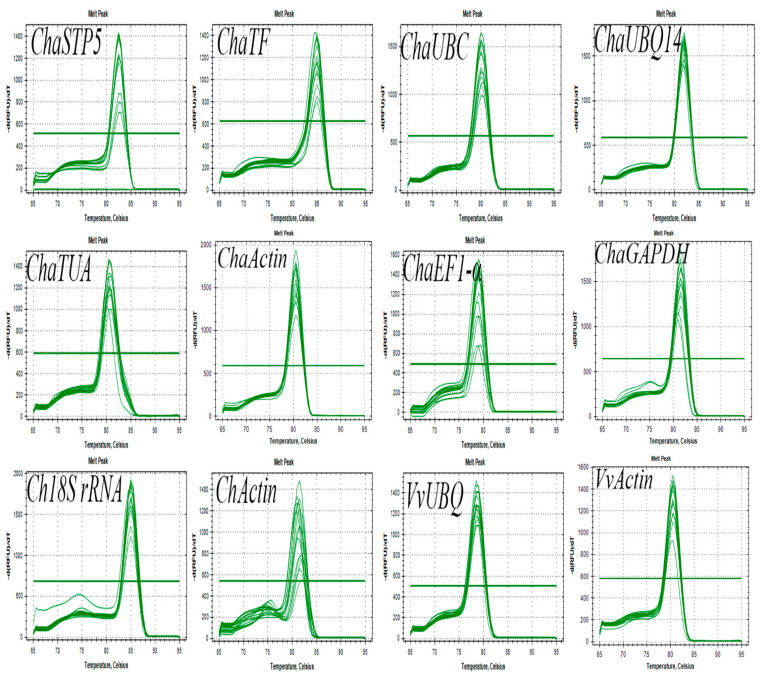
Melt curve analyses of RT-qPCR products for 12 candidate reference genes.

Agarose gel electrophoresis of the products amplified by RT-PCR revealed single fragments in all cases (Figure 1), demonstrating the high specificity of the primers. Amplification of the *ChActin* gene resulted in a lower molecular weight band, compared with the negative control group, which was found to be a primer dimer. Further, the expression of *ChaSTP5* and *ChaTF* differed among tissues. Accordingly, these preliminary data demonstrate that these two genes were not suitable as reference genes. Using the primer pairs designed for the 12 candidate reference genes, gene-specific amplification was also confirmed by the generation of a single peak on melting curve analyses following RT-qPCR (Figure 2).

### 2.2. Expression Levels of Selected Candidate Reference Genes

**Figure 3:**
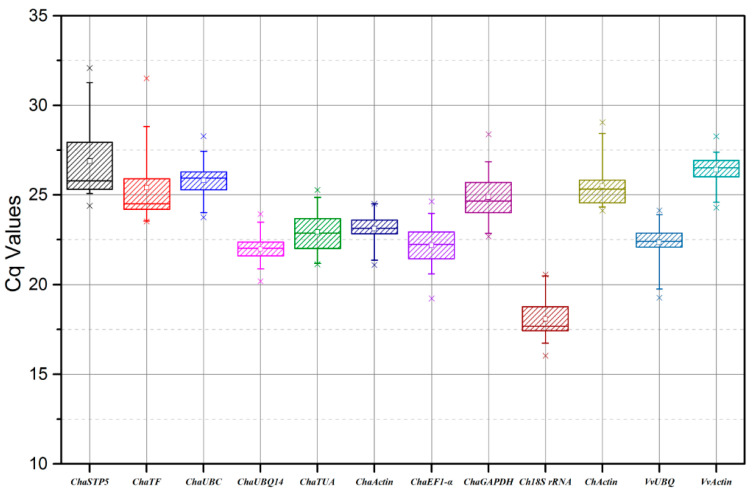
Cq values of candidate reference genes in 24 samples. Boxes indicate the interquartile range. Lines across the boxes depict the medians. Small white boxes within colored boxes indicate mean values. Whiskers represent the 95th and 5th percentiles. The × symbols denote minimum and maximum values.

When assessing a set of reference genes, the most straightforward method is to determine the range of Cq (quantification cycle) values and calculate the coefficient of variance. From the graph, the Cq value range for the 12 candidate reference genes in 24 samples was 16.02–32.09 (Figure 3), representing the highest and lowest accumulation levels, respectively, in different tissues, and indicating considerable variation. Cq values are inversely proportional to expression levels, hence the gene with the lowest level of gene expression across all 12 tested samples was *ChaSTP5* (Cq range, 24.39–32.09), and that with the highest was *Cha18S rRNA* (Cq range, 16.02–20.55); the other 10 candidate reference genes exhibited intermediate expression levels.

The mean Cq value, standard deviation (SD), and coefficient of variation (CV) of each candidate reference gene in all samples was analyzed using Excel (Table 3). According to the coefficient of variation, we made a preliminary assessment of the expression stability of the 12 candidate reference genes. *ChaSTP5* (8.33%) and *ChaTF* (8.05%) had the worst stability, *VvActin* (3.34%) and *ChaActin* (3.48%) had the highest stability, and the stability of the remaining candidate reference genes was ordered as follows: *ChaUBQ14 > ChaUBQc > VvUBQ > ChActin > ChaTUA > ChaGAPDH > ChaEF1-α > Cha18s-rRNA.*

### 2.3. Expression Stability of Candidate Reference Genes

A simple comparison of raw Ct (threshold cycle) values is insufficient to evaluate the expression stability of candidate reference genes. To minimize bias, a more accurate assessment is required; therefore, we applied four different commonly-used algorithms, geNorm [31], NormFinder [32], BestKeeper [33], and Delta Ct [24], to rank the expression stabilities of the 12 reference genes across all experimental sets.

#### 2.3.1. geNorm Analysis

For relative comparison, geNorm transforms raw Ct values into quantities, and calculates a gene expression stability measure, M (where a lower average value of M indicates more stable expression), for each gene, based on the average pairwise expression ratio between it and each of the other genes under study. The candidate gene is considered unsuitable as a reference gene if it exceeds a threshold of M > 1.5, as suggested by geNorm.

**Figure 4:**
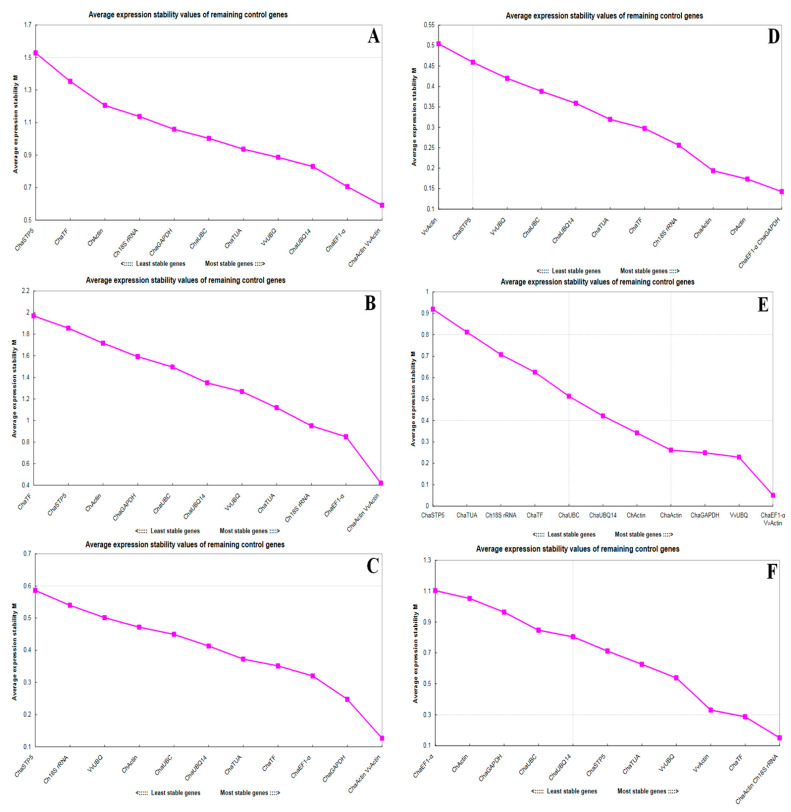
Expression stability (M) of twelve reference genes across different sample groups calculated using geNorm. M values of reference genes (**A**) in 24 sample groups, (**B**) in different tissues, (**C**) at different times after pollination, (**D**) in styles at 4 h after cross/self-pollination, (**E**) at different stages of flowering, and (**F**) in male catkins at different stages of elongation.

In all cases, the M values were <1, indicating that the selected genes all had relatively acceptable expression stability values. Nevertheless, it is likely better to choose different reference genes, depending on the samples to be studied. In our study, geNorm selected the best reference genes for each sample group (Figure 4). The two candidate genes, *ChaActin* and *VvActin*, achieved high expression stability, with M < 0.7, in a total of 24 sample groups (Figure 4A), different tissues (Figure 4B), and at different times after pollination (Figure 4C). The M value was lowest for *ChaEF1-α* and *ChaGAPDH*, indicating that they were the most stably expressed gene pair, in styles at 4 h after cross/self-pollination (Figure 4D). When styles at different flowering stages (Figure 4E) were considered, *ChaEF1-α* and *VvActin* were the best candidate genes. Finally, *ChaActin* and *Cha18s rRNA* were the two most stable genes in male catkins at different stages of elongation (Figure 4F).

**Figure 5:**
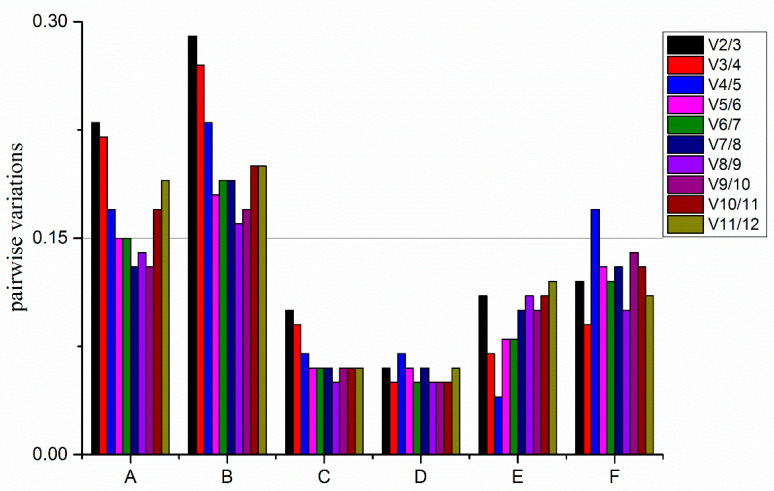
Determination of the optimal number of reference genes for different sample groups using geNorm. (**A**–**F**) represent different sample groups. (**A**) in 24 sample groups, (**B**) in different tissues, (**C**) at different times after pollination, (**D**) in styles at 4 h aftecross/self-pollination, (**E**) at different stages of flowering, and (**F**) in male catkins at different stages of elongation.

The optimal number of reference genes could also be determined using geNorm, according to the pairwise variation value (Vn/n + 1). The default cut-off threshold of 0.15 was applied, where a Vn/Vn + 1 value < 0.15 indicates that n reference genes would be needed for accurate normalization, without introducing another (n + 1) reference gene. The V2/3, V3/4, and V4/5 values for all sample groups (Figure 5A) were > 0.15, while V5/6 was 0.145 (<0.15), suggesting that five reference genes (*ChaActin*, *VvActin*, *ChaEF1-α*, *ChaUBQ14*, and *VvUBQ*) would be adequate for the normalization of our RT-qPCR, and an additional reference gene was not required; however, gene normalization in four sample groups (styles at different times after self-pollination, styles at 4 h after cross/self-pollination, styles at different flowering stages, and male inflorescence at different elongation stage) (Figure 5C–F) required the use of only two reference genes, since the V2/3 value was <0.15. When analyzing different tissues (Figure 5B), Vn/Vn + 1 values were >0.15; however, according to the instruction of geNorm^TM^ housekeeping gene selection kit with perfect Probe^TM^ (Primerdesign Ltd. Nursling Street, Rownhams, Southampton, SO16 0AJ, United Kingdom), the 0.15 threshold is not a strict restriction.

#### 2.3.2. NormFinder Analysis

The NormFinder program is a Microsoft Excel-based Visual Basic application that determines the expression stabilities of reference genes by ranking all candidate reference genes, based on intra- and intergroup variation for normalization factor calculations, and combining both results into a stability value for each candidate reference gene. This approach avoids misinterpretation caused by artificial selection of co-regulated genes. 

We applied NormFinder to calculate stability values (SV), to determine the stability of the candidate reference genes, where SV was lower for more stably expressed genes. Stability values for each gene in the six sample groups are presented in Table 4. *ChaActin* was the best reference gene in all the sample group (SV = 0.410), as well as the most stably expressed gene in different tissues (SV = 0.357), and at different times after self-pollination (SV = 0.121). For the cross- and self-pollination group (SV = 0.038), and the styles group (SV = 0.017), *ChaEF1-α* had the most stable expression and was the ideal reference gene. Both *ChaActin* and *Cha18s rRNA* were good reference genes in male inflorescences (SV = 0.052).

#### 2.3.3. BestKeeper Analysis

BestKeeper identifies the most stably expressed genes, based on three variables: standard deviation (SD), percentage covariance (CV), and coefficient of correlation (r). In the BestKeeper package, the best reference genes are those that are most stable and have the lowest CV ± SD (SD < 1) and the highest r [34]. 

BestKeeper can only analyze ten candidate reference genes simultaneously; therefore, we analyzed all reference genes, except for *ChaSTP5* and *ChaTF*, which had the least stable original Cq values. The variations (SD and CV) in data for all candidate reference genes were subject to preliminary analysis, and the results (Table 5) showed that all tested genes had SD values < 1 in all samples group, indicating that they were suitable candidate reference genes. SD values > 1 were generated for seven candidate reference genes (*ChaUBC*, *ChaTUA*, *ChaEF1-α*, *ChaGAPDH*, Cha18srRNA, *ChActin*, *VvUBQ*) in different tissues, one candidate reference gene (*ChaTUA*) in styles at different stages of flowering, and three candidate reference genes (*ChaEF1-α*, *ChaGAPDH*, *ChActin*) in male inflorescences at different stages of elongation, disqualifying them as reference genes. Analysis of Pearson correlation coefficient (r) values showed that the top candidate genes, with r > 0.9, were as follows: *ChaActin* was the most stable gene in groups (A, B, C, D, and F), except for group E; while in group E, the order of stability is *VvActin*, *VvUBQ*, *ChaGAPDH*, *ChaEF1-α*, *ChaActin*, *ChaTUA*, and *ChActin*. Although *ChaActin* was relatively in a lower-ranking, it has a very high r value.

#### 2.3.4. Delta Ct Analysis

The principle of the Delta Ct algorithm is similar to that of geNorm; both are based on Cq values and follow a pairwise approach, but with different procedures and outcomes [35]. The progressive exclusion of genes by geNorm increases the tendency to select the most correlated genes, whereas the Delta Ct method compares the relative expression of pairs of genes within each sample, to confidently identify useful reference genes [36].

As shown in Table 6, the results of Delta CT analyses largely agreed with that of geNorm, recommending that the most stable gene in sample groups A, B, and C was *ChaActin*. While *VvActin* and *Cha18s rRNA* were the optimal candidate reference genes in sample groups E and F, respectively. In sample group D, *ChaActin*, *ChaEF1-α* were the three most stable genes.

#### 2.3.5. Comprehensive Evaluation of the Stability of Candidate Reference Genes in 24 Samples 

To determine the optimal reference genes, The RefFinder approach (https://www.heartcure.com.au/reffinder/) was used to determine the comprehensive rankings of candidate reference genes based on the results of four common analysis programs (geNorm, NormFinder, BestKeeper, and Delta Ct) [37]. The comprehensive ranking of RefFinder was shown in Table 7, *ChaActin* and *VvActin* were selected as the most suitable reference genes. At the same time, the most suitable reference gene for each group of samples selected from different software packages as shown in Table 8. *ChaActin* and *ChaEF1-α* were most stable in different times after self-pollination (Sample group C) and 4 h after self- and cross-pollination (sample group D), respectively. Both of them were considered as the most suitable reference genes for pollen-pistil interaction in *Corylus*.

### 2.4. Validation of Selected Candidate Reference Genes

To further evaluate the reliability of the top two reference genes (*ChaActin* and *ChaEF1-α*), we selected *CavPrx* (*Corylus avellana* peroxidase) as a target gene for RT-qPCR amplification to validate the normalization efficiency of the selected reference genes. 

**Figure 6:**
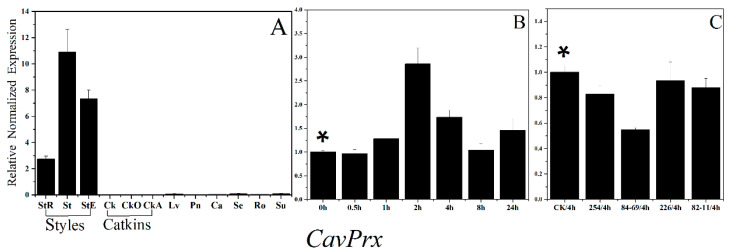
Expression analysis of the target gene, *CavPrx*, using *ChaActin* and *ChaEF1-α* as reference genes. * Control samples. The expression level of *CavPrx* (**A**) in different tissues, (**B**) at different times after self-incompatible pollination, (**C**) at 4 h after different affinity pollination. StR, styles at red-dot stage; St, blooming styles; StE, styles at the end-flower stage; Ck, catkins before elongation; CkO, catkins during elongation; CkA, catkins after elongation; Lv, young leaves; Pn, pollen; Ca, cambium of annual branch; Se, green stem; Ro, root tip; Su, sucker; 0 h, self-pollination 0 h; 0.5 h, self-pollination 0.5 h; 1 h, self-pollination 1 h; 2 h, self-pollination 2 h; 4 h, self-pollination 4 h; 8 h, self-pollination 8 h; 24 h, self-pollination 24 h; CK/4 h, non-pollination 4 h; 254/4 h: incompatible pollination 4 h with 84–254; 84–69/4 h; incompatible pollination 4 h with 84–69; 226/4 h, compatible pollination 4 h with 84–226; 82–11/4 h, compatible pollination 4 h with 82–11.

Our result showed that the *CavPrx* expression in mature styles was three-fold more abundant than that in immature styles and almost absent in leaves and three different developmental stages of catkin (Figure 6A). Moreover, we also found out that the expression level of *CavPrx* in two compatible pollinations at 4 h was higher than in two incompatible pollinations at 4 h (Figure 6C). Our results are consistent with the findings reported by Beltramo et al. After self-pollination (Figure 6B), the expression level of *CavPrx* showed an upward–downward–upward trend, reaching a maximum value at 2 h. In summary, *ChaActin* and *ChaEF1-α* are suitable reference gene to analyze the expression of related genes involved in pollen–pistil interaction. 

## 3. Discussion

RT-qPCR is an established method for gene expression analysis because of its high sensitivity, specificity, and reproducibility [38,39]; however, the bias caused by the RNA extraction, cDNA reverse transcription, and RT-qPCR processes can easily influence the final results of RT-qPCR [40]. To obtain more reliable experimental results, it is essential to introduce one or more reference genes for normalization [41]. According to previous research, an ideal reference gene should be stably expressed in all tissues, developmental stages, and physiological conditions, and may differ from the reference genes are suggested for use under other conditions [42]. Improper use of reference genes may lead to difficulties in detecting minor differences in gene expression or even erroneous or contrary conclusions [43]. Therefore, the most suitable reference genes, that show constant expression profiles, should be selected for specific experimental conditions and materials [44].

The geNorm, NormFinder, Delta Ct, BestKeeper, and RefFinder methods are commonly-used to evaluate the stability of reference gene expression, and none is currently considered superior to the others. Some reports have suggested that applying different analysis software can result in different validation results in the same tissue or treatment, due to their distinct statistical algorithms and analytical procedures [18]. To guarantee a comprehensive comparison, candidate reference genes included in this research containing traditional reference genes and reference genes obtained by analysis of transcriptome data. Five different statistical approaches, geNorm, NormFinder, Delta Ct, BestKeeper, and RefFinder were used to evaluate the expression stability of the 12 candidate reference genes.

The BestKeeper method offers an apparent solution to measurement of stability, since it uses standard deviation as a direct measure of variation; however, this means that the only condition applied is that the genes under evaluation are not co-regulated. In this case, a gene could show a low standard deviation while still being a good reference gene. Nevertheless, it is risky to assume that genes are not co-regulated, as this cannot be easily demonstrated. The geNorm and the comparative Delta Ct approaches rank genes mainly according to their correlation; these methods tackle the same problem; that is, they select the most stable genes by assuming “that the control reference genes are not co-regulated”. Consequently, selection of two co-regulated genes could ruin the analysis, leading to selection of unsuitable reference genes [31]. Finally, NormFinder takes into account intergroup variation, which should be as low as possible for a good reference gene; hence, it is not affected by the drawbacks of analysis of gene co-regulation. Therefore, the advantages and disadvantages of each algorithm should be considered when analyzing presumed reference genes, according to the experimental scheme. NormFinder and geNorm are the most extensive methodologies for finding optimum reference genes. In most cases, NormFinder and geNorm generate very similar results and this was confirmed in our experiments; the selection of optimal reference genes in all sample groups using NormFinder was in good agreement with those selected using geNorm. 

The aim of our study was to select the suitable reference genes for self-incompatibility response in *Corylus*. We performed a combined ranking of the 12 candidate reference genes in all samples, based on the RefFinder. *ChaActin*, *VvActin, ChaUBQ14,* and *ChaEF1-α* were selected as suitable reference genes. Then we analyzed the most suitable reference gene for each group of samples using four different software packages. As shown in Table 8, we found that *ChaActin* was the most suitable reference gene in most sample groups (A, B, C and F), *ChaEF1-α* exhibits excellent stability in sample group D and E by geNorm and NormFinder. Moreover materials from sample group C and E are involved in pollen-pistil interaction. Therefore, we selected *ChaActin* and *ChaEF1-α* as appropriate reference genes from our study, which could be used for the subsequent analysis of the expression of self-incompatibility (SI)-related genes.

In *Corylus*, previous research by Beltramo et al. demonstrated that CavPrx expression levels in mature styles were threefold those in immature styles, while it was almost absent in leaves and catkins. Moreover, the expression was higher (by almost 25%) in styles pollinated with compatible pollen than in incompatible pollinated styles [28]. In analysis of reference gene validation, we found the similar expression trend with Beltramo’s study is that the CavPrx expression of compatible pollinations was higher than that of incompatible pollinations. However the numerical value of increased expression of CavPrx was different from that in Beltramo’s study. We think this difference is due to the different pollinators. This regulation pattern could be noticed also for stigma-specific peroxidase (SSP) of *Senecio squalidus*, which expression was detected exclusively in the stigmas and increased with flower development, reaching a maximum level in mature stigmas [45]. This expression level of peroxidase gene suggests a possible involvement in pollen–pistil interaction; however, its possible function(s) remains to be further discussed.

*Actin* is also often used as a reference gene in plant species; for example, *Actin* was considered as a suitable reference gene for analyzing mRNA expression levels in different organs and at different development periods in *Lycoris sprengeri* comes ex Baker flowers and different *Lycoris radiata* (L’Her.) Herb. hybrids bulbs [46]. Wang et al. showed that *Actin* was the best reference gene for analysis of target gene expression levels in carrot (*Daucus carota*) roots and leaves at all five developmental stages [47]. Further, Fang et al. suggested that *Actin* was a suitable reference gene for RT-qPCR in plants infected with Rice Stripe Virus (RSV) and Rice Black Streaked Dwarf Virus (RBSDV) [48]. In addition, *EF1-α* is often used as reference gene. Zhang et al. used *EF1-α*/*18S rRNA* as reference gene to analyze the levels of farnesyl pyrophosphate synthetase (FPS) gene transcription in *Dendrobium officinale* [49]. Further, Li et al. evaluated the expression stability of eight candidate reference genes in different organs, during developmental stages, and under seven treatments in *Achyranthes bidentata* BI., and EF1-α was verified as a suitable reference gene across all tested samples [50]. However, previously published researches have stated that the ideal stable reference genes do not exist and the stability of these reference genes or a certain reference gene varied among various plant species and even changed under different experimental treatments in same species [19]. Chen have proved that *ChActin* (*Actin* from *C. heterophylla* Fisch.) was suitable as a reference gene for semi-quantitative PCR of hazelnut in different organs (bark, flower buds, catkins, and seed) [30]. However, in our study, the stability of *ChaActin* was superior to that of *ChActin*, because *ChActin* was not expressed in some particular tissue (blooming styles, catkins before elongation, leaves, and pollen) that involved in our experimental materials. Meanwhile, this result also indicated that reference genes obtained from primers designed based on sequences of experimental materials were more stable and the acquisition of related sequences from research materials can accelerate reference gene screening.

As the principles underlying the four programs used in this study were not the same, the stability rankings of the 12 candidate reference genes generated differed among them, indicating that the four programs should be used for simultaneous analysis when choosing reference genes, to provide more reliable screening results. Growing evidence suggests that the selection of more than one reference gene can provide more accurate data in plant RT-qPCR analysis [51]. Our results suggest that the optimal reference genes vary among samples. Therefore, selecting a suitable combination of reference genes, according to the experimental conditions and materials used, is necessary.

Except for the using of traditional reference genes and their homologue-sequences, it is also important to screen suitable reference genes from transcriptome database. To date, many researchers used gene expression databases, such as transcriptome data, to rapidly and efficiently screen for stable reference genes. For example, *UBC9* and *TUB* were identified as the most appropriate reference genes for RT-qPCR normalization in a hyper accumulating ecotype of *Sedum alfredii*, based on transcriptome data [52]. Zhu et al. selected *ACTB* and *UBCE* as reference genes during abscisic acid (ABA) treatment and dormancy transition, based on Chinese cherry flower bud transcriptome data [53]. Liu et al. assessed 22 candidate reference of tree peony including 16 new reference genes form transcriptome data and six traditional reference genes. Four newly reference genes (*PUF1639*, *MBF1A*, *PP2CFP* and *RPS9*) were selected and were superior to traditional ones in terms of their expression stability [54].

## 4. Materials and Methods

### 4.1. Plant Materials and Pollination Treatments

In this study, the Ping’ou hybrid hazelnut “*Dawe*i” (breeding code name: 84–254), “*Liaozhen No. 3*” (breeding code name: 84–226), “*Liaozhen No. 7*” (breeding code name: 82–11), and “*Liaozhen No. 9*” (breeding code name: 84–69) cultivars were used as samples. Trees were grown in Yuquanshan hazelnut experimental field at the Chinese Academy of Forestry.

An appropriate amount of one-year-old branches (approximately 60–80 cm long) were collected from all cultivars grown in the field in early spring, 2017, before the red dot stage of the stigmatic style (there are no complete flowers in hazelnuts; a cluster of red stigmatic styles extrudes from the tips of female flowers during anthesis), based on the phenological period. “*Dawei*” branches were emasculated, cleaned, and stored in a cold storage at 0 °C–2 °C for subsequent style collection and pollination. Branches from “*Dawei*”, “*Liaozhen No. 3*”, “*Liaozhen No. 7*”, and “*Liaozhen No. 9*”, with abundant well-developed male inflorescences were collected, cleaned and water-cultured in the greenhouse at 25 °C, with segregation. After the catkins elongated and pollen shed, pollen was collected directly from the catkins and stored in cotton-stoppered vials in a −80 °C freezer. As well as “*Dawei*” branches, catkins samples were also collected before elongation, at the beginning of elongation, and after elongation, for the experiment. 

Based on the results of a previous experiment, 30 stigmatic styles per sample were required for RNA extraction. Emasculated “*Dawei*” branches were removed from cold storage and randomly grouped into twelve groups (>15 branches per group) and then water-cultured in greenhouses at 25 °C (humidity, 60%). One group of branches was used for sampling styles at different stages of blooming. When the female flowers were at the full blooming stage, styles from seven groups of branches were pollinated with the pollen of “*Dawei*” and collected 0, 0.5, 1, 2, 4, 8, and 24 h after pollination; the styles from another three groups of branches were pollinated with pollen from “*Liaozhen No. 3*”, “*Liaozhen No. 7*”, and “*Liaozhen No. 9*” and collected 4 h after pollination. The styles from the last group were treated as controls and collected after 4 h, mature styles were dissected from the female flowers, immediately immersed in liquid nitrogen, and stored at −80 °C. 

The samples of different tissues listed in Table 9 were collected in the late spring of 2017. The cambium of annual branches and root suckers were sampled in the field, while the young leaves, green stems, and root tips were sampled from the tissue cultured saplings. 

A total of 24 samples (A) were collected in this study, including different tissues (B), “*Dawei*”styles at different times after self-pollination (C); different styles at 4 h after cross/self-pollination (D); styles at different flowering stages (E); and male catkins at different elongation stages (F) (Table 9). After collection, all samples were immediately immersed in liquid nitrogen and stored at −80 °C for subsequent RNA extraction. 

### 4.2. Candidate Reference Gene Selection and Primer Design

In our previous study, a set of style transcriptome data were obtained during compatible/incompatible pollen-stigma interactions of the Ping’ou hybrid hazelnut “*Dawei*” (unpublished data). Using this transcriptome data, new candidate reference genes were screened based on False Discovery Rate (FDR) and Log2 ratio of gene expression values; threshold used were FDR < 0.01 and Log2 ratio between −1 and 1. Using the same screening criteria that selected for new candidate reference genes, homologous sequences of seven traditional reference genes, including *Actin*, *Ubiquitin*, *18S ribosomal RNA*, *alpha tubulin*, *beta-tubulin*, *elongation factor 1-alpha*, and *GAPDH*, were selected from the transcriptome data. These sequences were listed in the supplementary material. Further, some published reference genes (*ChActin* [12], *VvUBQ*, *VvActin* [28], and *Cha18S rRNA* [29]) of hazelnuts were also used as the candidates genes for evaluation in this study. 

RT–qPCR primers were designed for all genes using the Primer 3 program (http://bioinfo.ut.ee/primer3-0.4.0/). To ensure maximum specificity and efficiency during PCR amplification, a stringent set of criteria were applied for primer design [55,56], including optimal melting temperatures (Tm) of 58 °C–62 °C, lengths of 19–21 nucleotides, guanine and cytosine (GC) content of 45%–55%, and PCR amplicon lengths of 150–200 bp. These primers were analyzed for specificity using the NCBI Basic Local Alignment Search Tool (BLAST) program (http://www.ncbi.nlm.nih.gov/BLAST/). All primer sequences are presented in Table 10 and were synthesized by Beijing Liuhe Huada Gene Technology Co., Ltd. 

### 4.3. Total RNA Extraction and cDNA Synthesis

Total RNA was extracted from samples using the cetyltrimethylammonium bromide (CTAB) method [57]. RNA integrity was evaluated by electrophoresis in 1% agarose gel and staining with GelRed Nucleic Acid Gel Stain 10,000× in water (Biotium, Fremont, CA, USA). A NanoDrop 8000 spectrophotometer (Thermo, Wilmington, DE, USA) was then used to determine the purity and concentration of the RNA samples. Total RNA samples (1 μg) were reverse transcribed to generate cDNA using an iScript™ cDNA synthesis kit (Bio Rad, Hercules, CA, USA) in a total volume of 20 μL, according to the manufacturer’s protocol. cDNA preparations were diluted 5-fold with nuclease-free deionized water (Tiangen, Beijing, China) for use as template in PCR analysis.

### 4.4. RT-PCR and RT-qPCR Analysis

RT-PCR was performed in 25 μL, containing 12.5 μL 2× TSINGKE Master Mix (TSINGKE, Beijing, China), 1 μL of each primer F (10 μM) and R (10 μM), 1 μL of cDNA, and 9.5 μL of PCR-grade water, in a Veriti 96-well thermal cycler (Applied Biosystems, Foster City, CA, USA), with the following steps: 94 °C for 3 min; 35 cycles of 94 °C for 30 s, Tm for 45 s, 72 °C for 30 s; then 72 °C for 10 min and maintained at 4 °C. PCR products were separated by 1.5% agarose gel electrophoresis and visualized with GelRed Nucleic Acid Gel Stain 10,000× in water (Biotium, USA).

RT-qPCR amplification was performed using a CFX 96TM Real-Time system (Bio Rad, USA), with iTaq™ Universal SYBR^®^ Green Supermix (Bio Rad, USA). Each PCR reaction was performed in a 20 μL volume, containing: 4 μL cDNA, 10 μL iTaq Universal SYBR Green Supermix (2×), 0.5 μL of each10 μM Primer F and R, and 5 μL PCR-grade water. Thermal cycler parameters were as follow: 3 min at 95 °C; then 40 cycles of 5 s at 94 °C, 30 s at 56 °C. Melting curve analysis was carried out from 65 °C to 95 °C to evaluate the specificity of the PCR products. Cq (quantification cycle) values were automatically determined and each PCR reaction was repeated three times (technical replicates) per sample. No-template controls were included. The PCR programming comprised an initial denaturation at 95 °C for 30 s, followed by 40 cycles of denaturation at 95 °C for 3 s and primer annealing and extension at 56 °C for 30 s, with fluorescent signal recording. Subsequently, a melting curve analysis was carried out from 65 °C to 95 °C to evaluate the specificity of the PCR products. Five serially diluted cDNA samples were used as template to construct standard curves for each primer pair, where RT-qPCR composition and conditions were as described above. Standard curves were constructed by linear regression, based on Cq values for all dilution points in a series, using CFX Manager 3.1 software (Bio Rad, Hercules, CA, USA). Correlation coefficient (R2) and slope values were obtained from the standard curve, and corresponding PCR amplification efficiencies (E) calculated, according to the following equation: E = 10(−1/slope). 

### 4.5. Data Analysis

Statistical analysis of Cq values was conducted using Microsoft Excel 2010. Ct values were transformed into relative quantities (Q values) using qBase [16,58,59] with the following formula:

Q = E Cq min − Cq sample

where ‘E’ indicates the PCR amplification efficiency, Cq min the minimum Cq value of all samples in one group, and Cq sample the mean of Cq values for all samples in one group. Four Excel-based software tools, geNorm, NormFinder, BestKeeper, and Delta Ct, were used to evaluate the expression stability of all candidate reference genes. Moreover, optimal numbers of reference genes required for gene expression normalization were calculated using geNorm. Finally, RefFinder, a web-based tool, provided a comprehensive ranking of selected reference genes.

### 4.6. Reference Gene Validation by Analysis of Class III Peroxidase Gene Expression

In this study, *CavPrx* [28] was used as a target gene to assess the reliability of the top two potential reference genes, *ChaActin* and *ChaEF1-α*. The relative expression level of CavPrx at different style flowering stages was determined and normalized using the RT-qPCR conditions described above. Relative expression levels were calculated using the 2^−ΔΔCt^ method.

## 5. Conclusions

Stably expressed reference genes are necessary to accurately understand the mechanisms underlying self-incompatibility and identify appropriate SI-related genes. There have been few reports on the search for reference genes for study of hazelnut self-incompatibility. Therefore, we used four different statistical programs and tested the expression stability of 12 selected candidate genes in six sample groups. Our results revealed that *ChaActin* and *ChaEF1-α* were the most stably-expressed reference genes for pollen–pistil interaction. These results will benefit future gene expression studies of self-incompatibility, and other genetic research, in *Corylus*.

## Figures and Tables

**Table 1:** Eight candidate reference genes.

No	Gene ID	Description	Gene	Identity %
1	Unigene27057_All	Transcription elongation factor SPT5	*ChaSTP5*	*——*
2	Unigene8224_All	Homeodomain-containing transcription factor	*ChaTF*	*——*
3	Unigene376_All	Ubiquitin C	*ChaUBC*	*——*
4	Unigene17976_All	Polyubiquitin 14 (*Arabidopsis thaliana*, NM_001125450.1a)	*ChaUBQ14*	84.39%
5	CL4792.Contig2_All	α-tubulin (*Betula pendula*, FJ228477.1a)	*ChaTUA*	90.23%
6	CL750.Contig12_All	*Actin* (*Betula pendula*, EU588981.1a)	*ChaActin*	97.35%
7	CL828.Contig4_All	*EF1-α* (*Arabidopsis thaliana*, NM_001035916.2a)	*ChaEF1-α*	87.90%
8	Unigene22682_All	*GAPDH* (*Populus trichocarpa*, FN396887.1a)	*ChaGAPDH*	91.53%

No. 1–3, new reference genes, selected from transcriptome data. No. 4–8, Sequences homologous to traditional reference genes from the transcriptome data. Species and National Center for Biotechnology Information (NCBI) Accession number of traditional reference gene.

**Table 2:** Primers for candidate reference genes.

Gene	Length (bp)	Efficiencies (E %)	R^2^	Reference
*ChaSTP5*	179	109.4	0.99	This study
*ChaTF*	192	91.9	0.997	This study
*ChaUBC*	155	107.1	0.986	This study
*ChaUBQ14*	197	92.8	0.995	This study
*ChaTUA*	155	97.5	0.995	This study
*ChaActin*	179	91.7	0.993	This study
*ChaEF1-α*	175	86.3	0.995	This study
*ChaGAPDH*	164	88.3	0.982	This study
*Cha18s rRNA*	277	100.8	0.988	[29]
*ChActin*	101	118.1	0.984	[30]
*VvUBQ*	99	93.2	0.997	[28]
*VvActin*	100	121.6	0.994	[28]

**Table 3:** Statistical analysis of candidate reference gene Cq values.

Gene	Mean	SD	CV
*ChaSTP5*	26.89	2.24	8.33%
*ChaTF*	25.41	2.04	8.05%
*ChaUBQc*	25.82	0.99	3.82%
*ChaUBQ14*	21.97	0.8	3.66%
*ChaTUA*	22.93	1.17	5.10%
*ChaActin*	23.13	0.8	3.48%
*ChaEF1-α*	22.19	1.22	5.50%
*ChaGAPDH*	24.87	1.33	5.36%
*Cha18s rRNA*	18.07	1.11	6.15%
*ChActin*	25.5	1.26	4.94%
*VvUBQ*	22.36	1.08	4.83%
*VvActin*	26.4	0.88	3.34%

**Table 4:** Stability values for candidate reference genes generated using NormFinder.

Gene	Group					
	A	B	C	D	E	F
*ChaSTP5*	1.546	1.663	0.510	0.398	0.968	0.671
*ChaTF*	1.157	1.408	0.122	0.248	0.584	0.263
*ChaUBC*	0.869	1.171	0.269	0.298	0.326	0.875
*ChaUBQ14*	0.589	0.746	0.254	0.234	0.134	0.827
*ChaTUA*	0.451	0.627	0.248	0.275	0.927	0.425
*ChaActin*	0.410 *	0.357 *	0.121 *	0.064	0.278	0.052 *
*ChaEF1-α*	0.461	0.539	0.270	0.038 *	0.017*	0.898
*ChaGAPDH*	0.838	1.205	0.248	0.057	0.405	0.743
*Cha18s rRNA*	0.557	0.697	0.396	0.246	0.665	0.052 *
*ChActin*	0.722	0.971	0.352	0.129	0.367	0.866
*VvUBQ*	0.683	0.806	0.401	0.364	0.359	0.429
*VvActin*	0.582	0.393	0.215	0.468	0.047	0.371

* indicates the most stable candidate reference genes for each sample group.

**Table 5:** Stability analysis of 10 candidate reference genes using BestKeeper.

Group	Index	Gene									
		*ChaUBC*	*ChaUBQ-14*	*ChaTUA*	*ChaActin*	*ChaEF1-α*	*ChaGAPDH*	*Cha18srRNA*	*ChActin*	*VvUBQ*	*VvActin*
A	r	0.39	0.57	0.77	0.84 *	0.91 *	0.59	0.47	0.57	0.58	0.77
	SD	0.73	0.59	0.91	0.56	0.91	0.99	0.87	0.90	0.69	0.65
	CV (%)	2.82	2.68	3.99	2.43	4.11	3.99	4.82	3.53	3.07	2.45
B	r	0.32	0.44	0.76	0.92 *	0.91 *	0.44	0.74	0.56	0.69	0.85
	SD	1.20	0.83	1.47	0.82	1.48	1.46	1.11	1.64	1.13	0.86
	CV (%)	4.65	3.78	6.33	3.59	6.66	5.77	5.97	6.27	5.22	3.28
C	r	0.70	0.90	0.88	0.91	0.97*	0.94 *	0.33	0.86	0.61	0.83
	SD	0.37	0.49	0.53	0.40	0.61	0.59	0.39	0.54	0.58	0.34
	CV (%)	1.44	2.21	2.31	1.68	2.78	2.37	2.26	2.17	2.55	1.30
D	r	−0.02	0.35	0.92	0.96 *	0.90	0.95 *	0.88	0.76	0.10	0.89
	SD	0.15	0.19	0.52	0.19	0.24	0.31	0.42	0.18	0.33	0.58
	CV (%)	0.59	0.87	2.31	0.81	1.08	1.26	2.36	0.70	1.47	2.16
E	r	0.67	0.89	0.92	0.94	0.99	0.99	0.08	0.95	0.99 *	1.00 *
	SD	0.11	0.39	1.36	0.74	0.59	0.89	0.56	0.69	0.80	0.48
	CV (%)	0.42	1.75	6.08	3.18	2.62	3.53	3.11	2.72	3.49	1.83
F	r	−0.87	−1.00	0.43	1.00 *	0.98	0.99*	0.97	0.97	0.16	0.92
	SD	0.43	0.37	0.15	0.53	1.54	1.35	0.51	1.19	0.18	0.72
	CV (%)	1.76	1.80	0.64	2.32	6.88	5.59	2.58	4.46	0.83	2.75

* indicates the most stable candidate reference genes for each sample group.

**Table 6:** Stability value (mean SD) rankings for 12 candidate reference genes according to Delta Ct analysis.

Group						
Rank	A	B	C	D	E	F
1	*ChaActin*	*ChaActin*	*ChaActin*	*ChaActin*	*VvActin*	*Cha18s rRNA*
	(1.20)	(1.43)	(0.47)	(0.37)	(0.67)	(0.82)
2	*ChaUBC*	*VvActin*	*ChaTF*	*ChaEF1-α*	*ChaEF1-α*	*ChaActin*
	(1.23)	(1.48)	(0.49)	(0.38)	(0.68)	(0.83)
3	*VvActin*	*Cha18s rRNA*	*VvActin*	*ChaGAPDH*	*ChaUBQ14*	*VvActin*
	(1.27)	(1.67)	(0.51)	(0.38)	(0.74)	(0.91)
4	*ChaEF1-α*	*ChaEF1-α*	*ChaTUA*	*ChActin*	*ChaActin*	*VvUBQ*
	(1.32)	(1.70)	(0.56)	(0.40)	(0.78)	(0.98)
5	*ChaTUA*	*VvUBQ*	*ChaUBC*	*ChaUBQ14*	*ChActin*	*ChaTF*
	(1.32)	(1.71)	(0.57)	(0.48)	(0.78)	(1.01)
6	*ChaUBQ14*	*ChaTUA*	*ChaUBQ14*	*ChaTF*	*ChaUBC*	*ChaTUA*
	(1.35)	(1.74)	(0.58)	(0.49)	(0.79)	(1.02)
7	*Cha18s rRNA*	*ChaUBQ14*	*ChaGAPDH*	*Cha18s rRNA*	*VvUBQ*	*ChaSTP5*
	(1.40)	(1.87)	(0.58)	(0.49)	(0.80)	(1.18)
8	*ChActin*	*ChActin*	*ChActin*	*ChaUBC*	*ChaGAPDH*	*ChActin*
	(1.45)	(2.01)	(0.58)	(0.51)	(0.87)	(1.22)
9	*VvUBQ*	*ChaUBC*	*ChaEF1-α*	*ChaTUA*	*ChaTF*	*ChaUBC*
	(1.48)	(2.17)	(0.62)	(0.51)	(1.05)	(1.25)
10	*ChaGAPDH*	*ChaSTP5*	*Cha18s rRNA*	*VvUBQ*	*Cha18s rRNA*	*ChaGAPDH*
	(1.65)	(2.32)	(0.69)	(0.61)	(1.14)	(1.33)
11	*ChaTF*	*ChaGAPDH*	*VvUBQ*	*VvActin*	*ChaTUA*	*ChaUBQ14*
	(2.04)	(2.40)	(0.71)	(0.62)	(1.40)	(1.34)
12	*ChaSTP5*	*ChaTF*	*ChaSTP5*	*ChaSTP5*	*ChaSTP5*	*ChaEF1-α*
	(2.29)	(2.72)	(0.81)	(0.65)	(1.43)	(1.55)

**Table 7:** Stability rankings of 12 candidate reference genes in all samples.

Gene	Ranking Order	Gene	Ranking Order	Gene	Ranking Order
*ChaActin*	1	*ChaTUA*	5	*ChActin*	9
*VvActin*	2	*VvUBQ*	6	*ChaGAPDH*	10
*ChaUBQ14*	3	*Ch18S rRNA*	7	*ChaTF*	11
*ChaEF1-α*	4	*ChaUBC*	8	*ChaSTP5*	12

**Table 8:** The most suitable reference gene for each group of samples selected from different software packages.

Samples/Algorithms	geNorm	NormFinder	BestKeeper	Delta Ct
A	*ChaActin*	*ChaActin*	*ChaActin*	*ChaActin*
B	*ChaActin*	*ChaActin*	*ChaActin*	*ChaActin*
C	*ChaActin*	*ChaActin*	*ChaActin*	*ChaActin*
D	*ChaEF1-α*	*ChaEF1-α*	*ChaActin*	*ChaActin*
E	*ChaEF1-α*	*ChaEF1-α*	*ChaActin*	*VvActin*
F	*ChaActin*	*ChaActin*	*ChaActin*	*Cha18s rRNA*

**Table 9:** Description of samples.

	Group	Sample	Description	Group		Sample	Description
A	B	St	Blooming styles	A	C	0 h	Styles, 0 h after self-pollination
		Ck	Catkins before elongation			0.5 h	Styles, 30 m after self-pollination
		Lv	Young leaves			1 h	Styles, 1 h after self-pollination
		Pn	Pollen			2 h	Styles, 2 h after self-pollination
		Ca	Cambium of annual branch			4 h	Styles, 4 h after self-pollination
		Se	Green stem			8 h	Styles, 8 h after self-pollination
		Ro	Root tip			24 h	Styles, 24 h after self-pollination
		Su	Root Sucker		D	CK/4 h	Styles, 4 h without pollination
	E	StR	Styles at red-dot stage			*” Dawei”*/4 h	Styles, 4 h after incompatible pollination with “*Dawe*i”
		St	Blooming styles			*” Liaozhen No. 9”*/4 h	Styles, 4 h after incompatible pollination with “*Liaozhen No.9*”
		StE	Styles at end-flower stage			*” Liaozhen No. 3”*/4 h	Styles, 4 h after compatible pollination with “*Liaozhen No.3*”
	F	Ck	Catkins before elongation			*” Liaozhen No. 7”*/4 h	Styles, 4 h after compatible pollination with “*Liaozhen No.7*”
		Ck0	Catkins at the beginning of the elongation				
		CkA	Catkins after elongation				

**Table 10:** RT-qPCR primer sequences.

Gene	Primer (5′-3′)	Temperature (°C)
*ChaSTP5*	F: ACCATTTCCGGATGTTTGAG	58
	R: GTCGCCCTTCTTACAGTTGC	
*ChaTF*	F: GTGCCTAGCCATCCTCATGT	60
	R: ATCACCCTGACATCCTCGTC	
*ChaUBC*	F: CAGGCTCGCCAATCTTACTC	54
	R: ACCCCCTTTTTCAGAAGCAT	
*ChaUBQ14*	F: CCTTGCATCTGGTGTTGAGA	60
	R: AGTACGCCCATCCTCCAAT	
*ChaTUA*	F: TCTCCACAGGTTTCCACCTC	60
	R: GTGTAGGTGGGTCGCTCAAT	
*ChaActin*	F: GAGCTGAGAGATTCCGTTGC	56
	R: AGCAATACCTGGGAACATGG	
*ChaEF1-α*	F: TTGCCTTTACCCTTGGTGTC	54
	R: TCGAAACCAGAGATGGGAAC	
*ChaGAPDH*	F: AGCTCGTCGCTGTTAACGAT	60
	R: GTTCCTGAAGCCGAAAACTG	
*Cha18s rRNA*	F: AGACACTCGTGCCTTCTTGCC	60
	R: CAACGATGCGTGACACCCAG	
*ChActin*	F: TGGTCAAGGCTGGGTTTGC	58
	R: CTGACCCATCCCAACCATGA	
*VvUBQ*	F: TCTGAGGCTTCGTGGTGGTA	60
	R: AGGCGTGCATAACATTTGCG	
*VvActin*	F: GCCCCTCGTCTGTGACAATG	56
	R: CCTTGGCCGACCCACAATA	
*CavPrx*	F: CTCGAGGGTTTGACGTTGTTG	60
	R: GCTTCAGCAGCAAGGGCTAGA	

## Data Availability

The data presented in this study are available in supplementary material.

## Supplementary Materials

The following are available online at https://www.mdpi.com/2223-7747/10/1/159/s1.
