# Supplementary material for: Selection and Validation of Reference Genes for Quantitative RT-PCR Analysis in Corylus heterophylla Fisch. × Corylus avellana L
Source: Plants (Basel). 2021 Jan 15;10(1):159. doi: 10.3390/plants10010159 (PMC7830083; doi:10.3390/plants10010159)
Supplement: Supplementary file 1 [file plants-10-00159-s001.pdf]

We obtained homologous sequences of 7 traditional reference genes based on transcriptome data using Blast.

The traditional reference genes and their homologous sequences are as follows

(1)*Actin*

>gi|182409984|gb|EU588981.1| Betula platyphylla actin mRNA, complete cds

GATTCTACACCATTTTCCTCTCTGCGTCCTCGGCCTCCTCTCAACTGAAACTCAGAGAAAGAGAGCCA
AGCCTTCGTCTTTGGAGGTCGGCTTGCTTTTCTGCGTCCTCGATCGTCTCATTTACGTCGTCGTTTTTGT
CATCTCAGATTTGTGAAAAATGGCCGATGCCGAGGATATTCAGCCCCTTGTTTGCGACAATGGAACTGG
GATGGTGAAGGCTGGTTTTGCTGGTGATGATGCTCCCAGGGCAGTATTTCCTAGTATTGTAGGTCGTCC
CCGTCACACTGGTGTTATGGTTGGGATGGGCCAAAAAGATGCTTATGTTGGTGATGAAGCCCAATCCA
AGAGGGGTATTCTTACCTTGAAATATCCAATTGAGCACGGCATTGTCAGTAATTGGGATGACATGGAAA
AGATCTGGCATCACACATTCTACAATGAGCTTCGTGTTGCTCCTGAAGAGCACCCAGTGCTTCTCACTG
AAGCTCCTCTCAACCCTAAGGCCAACAGAGAAAAGATGACTCAAATCATGTTTGAGACCTTCAATGTG
CCTGCCATGTATGTTGCTATCCAGGCTGTTCTCTCCCTGTATGCCAGTGGTCGTACAACTGGTATTGTGC
TGGATTCTGGTGATGGTGTGAGTCACACTGTGCCAATCTACGAAGGGTATGCCCTCCCACATGCCATCC
TCCGTCTGGACCTTGCTGGTCGTGATCTTACTGATGCGTTGATGAAGACTCTCACTGAGAGAGGGTATA
TGTTCACCACCACTGCCGAACGGGAAATTGTCCGTGACATGAAGGAGAAGCTTGCATATGTTGCCCTT
GACTATGAGCAGGAACTTGAGACTGCCAAGAGCAGCTCCTCTGTTGAGAAGAACTATGAATTGCCCGA
TGGACAAGTCATCACAATCGGAGCTGAGAGATTCCGATGCCCAGAAGTCCTCTTCCAGCCATCTCTGA
TCGGAATGGAAGCTGCTGGAATCCATGAGACCACCTACAACTCTATCATGAAATGTGACGTGGATATCA
GAAAGGATCTCTATGGAAACATTGTTCTTAGTGGTGGCTCAACCATGTTCCCTGGTATTGCTGACCGGA
TGAGCAAGGAGATCACTGCTCTTGCCCCAAGCAGCATGAAGATTAAGGTTGTGGCACCACCAGAGAG
GAAGTACAGTGTCTGGATCGGAGGATCAATCCTCGCATCCCTCAGCACCTTCCAGCAGATGTGGATTTC
CAAGGGTGAGTACGACGAGTCTGGTCCATCTATTGTCCACAGGAAGTGCTTCTAAATTCTACAGCAGT
GCTTTGATGGTGAGTTTTATTTCCATTTAGTTGGCTTTTCGTGTCATGTGTCATGTGAACTCAAAATTGG
TTGAAGTGGAAAGTGTTGAGGTAGGATCACTGAAGGAGGGTATGATATTTTGGTCTTGATATGTCCTCT
AGCTTTTCATCCGTGCGTGAGAGGCTTGCCTGTAGTTGTCCTCTGGATGTGGTGGGCAGAAAGCTGGT
CCTCCAAAGTCCTTCCTCGGGTTGCATTATCTGTGGTTCAACCATTCCCTTCAGTAGGATGTTTGTAGC
AGGAGAGTGATTGTGATGCTTTTTTTTTTATCCTTTCTTTTTTTTTTCTTTCTCTTCAATATTTGACGGTTT
TTTTTTTTTCCCTGGGAACATTAATGTTAATAGCTATTGTATGAGAAAATTTTATGTTAGTGTCAGTTTGC
GGTCATAAACTTTCTATGAAAACTATATTTAATCCAAAAAAAAAAAAAAAAAAAAAAAAAAA

> >CL750.Contig12_All

ATTTGGTCTAAATTTCATGTGCATTTCACAAATACCACTCGCTCTACAGCTATATATATACCCCTCTTTTCT
AGCTCTCTCTCCTATTCTACCCCATTTTCCTCTGCGTCCTCGGCCTCCTCTCAACTGATACTCAAGAGAT
AGAGAGCCAAGCCTTCGTCTTTGGAGGTCGGCTTGCTCTTCTTCGTCTTCGATCGCCTTATTTTCGTCG
TCTTTTTTGTAATCTCAGATTTGTGAAAAATGGCCGATGCCGAGGATATTCAGCCCCTTGTTTGTGACAA
TGGAACTGGAATGGTGAAGGCTGGTTTTGCTGGTGATGATGCTCCCAGGGCAGTGTTTCCTAGTATTGT
GGGTAGGCCCCGTCACACTGGTGTTATGGTTGGTATGGGCCAAAAAGATGCTTATGTTGGTGATGAAG
CTCAATCCAAGAGGGGTATTCTTACCTTGAAATATCCTATTGAGCATGGTATTGTCAGTAATTGGGATGA
CATGGAGAAGATCTGGCATCACACATTCTACAATGAGCTTCGAGTTGCTCCTGAAGAGCACCCAGTGC
TTCTCACTGAAGCTCCTCTCAACCCTAAGGCCAACAGAGAAAAGATGACTCAAATCATGTTTGAGACC
TTCAATGTGCCTGCCATGTATGTTGCTATCCAGGCTGTTCTCTCCCTGTATGCCAGTGGTCGTACAACTG
GTATTGTGCTGGATTCTGGTGATGGTGTGAGTCACACTGTGCCAATCTACGAAGGGTATGCCCTCCCAC

ATGCCATCCTTCGTTTGGACCTTGCTGGTCGTGATCTCACTGACGCTTTGATGAAGATTCTCACTGAGA
GAGGGTATATGTTCACCACCACTGCCGAACGGGAAATTGTCCGTGACATGAAGGAGAAGCTTGCATAT
GTTGCCCTTGACTATGAGCAGGAACTTGAGACTGCCAAGAGCAGCTCCTCTGTTGAGAAGAACTATGA
ATTGCCTGATGGACAAGTCATCACAATCGGAGCTGAGAGATTCCGTTGCCCAGAAGTCCTCTTCCAGC
CATCGCTGATTGGAATGGAAGCTGCTGGAATCCACGAGACCACCTACAACTCTATCATGAAATGTGAC
GTGGATATCAGAAAGGATCTCTACGGAAACATTGTTCTCAGTGGTGGCTCAACCATGTTCCCAGGTATT
GCTGACCGGATGAGCAAGGAGATCACTGCTCTTGCCCCAAGCAGCATGAAGATTAAGGTTGTGGCAC
CACCAGAGAGGAAGTACAGTGTCTGGATCGGAGGATCAATCCTTGCATCCCTCAGCACCTTCCAGCAG
ATGTGGATTTCAAAGGGCGAGTACGACGAGTCTGGTCCATCTATTGTCCACAGGAAGTGCTTCTAAATT
CTACAGCAGTGCTTTGATGCTTTTCATCTGTGCGTGAGAGGCTTACCTGTACTTGTCCTCTGGATGTGG
TGAGCAGAAAGCTGGTCCTCAAAAGTCCGTCCTCAGGTTGCATTATCTTGTGGTTCAACCATTCCCTTC
AGTAGGATGTTTGTAGCTGGAGAGTGATTGTGATGCTTCTTCTTTTTTTTTTTTTCCCTTTTTTTTTCTCC
TCCTCTTTCAATATTTGACGGTTTTTTTCCCTGGGAACATTAATGTTAATAGCTATTGTATGAGAAAATTT
TATGTTAGTGTCAGTTTGCGGTCATAAACTTTCTATGAAAACTATATTTAATTACTGGCTTGAAAGCTTC
TGAAGGATCCTTTGCCGCGCGCCCTCTGTTTTCTAATGATGTTTGTTGAATATTTATTCTCGGTTAGTGT
AATTTTTGGCTGGGTATGGATGTCTGATTCGTAATTAATTTAAAGAACAGTAAAATGCTATAGCCTACCT
AAGCCAGATCTGCCAATCAAAAGGGTTGTGCACCTCAAAAGGGTTATGTGGTCATTTACGGGAGTCGT
AGGAGGAAAAATAATATGACTACTATTTCTTCTGAATTTAACTTTGAAGGGGGATGG

(2) *UBQ14*

>gi|186511481|ref|NM_001125450.1| Arabidopsis thaliana polyubiquitin 14 mRNA, complete cds
TTCACAAATTCAGATTCCAAATTTCTCAAACTCTGAAATCGTTCTTTCAAATCTCTCAATCGGGATCAA
GATGCAGATCTTTGTTAAGACTCTCACCGGAAAGACTATCACCCTCGAGGTGGAAAGCTCTGACACCA
TCGACAACGTTAAGGCCAAGATCCAGGATAAGGAAGGCATTCCTCCGGATCAGCAGAGGCTTATTTTC
GCCGGAAAACAGCTAGAGGATGGCCGTACGTTGGCTGATTACAATATCCAGAAGGAATCCACCCTCCA
CTTGGTTCTCAGGCTCCGTGGTGGTATGCAGATTTTCGTCAAGACCTTAACGGGAAAGACGATTACTCT
TGAGGTGGAGAGTTCTGACACCATCGACAACGTCAAGGCCAAGATCCAAGACAAGGAGGGTATTCCT
CCGGACCAGCAGAGGTTGATCTTCGCTGGAAAGCAGTTGGAGGATGGCAGAACTCTTGCTGACTACA
ACATCCAGAAGGAGTCCACACTTCATCTTGTTCTCAGGCTCCGTGGTGGTATGCAGATCTTTGTCAAGA
CGTTGACTGGAAAGA
CTATCACTTTGGAGGTGGAGAGCTCTGACACTATCGACAATGTCAAAGCCAAGATCCAGGACAAAGA
GGGTATCCCACCGGACCAGCAGAGATTGATCTTCGCCGGAAAACAACTTGAAGATGGTAGAACTTTGG
CTGACTACAACATTCAGAAGGAGTCTACACTTCACTTGGTGTTGCGTCTCCGTGGAGGTATGCAGATTT
TCGTGAAGACTCTCACTGGAAAGACCATTACTCTTGAAGTTGAGAGCTCCGACACCATTGACAACGTG
AAGGCTAAGATCCAGGACAAGGAAGGTATCCCTCCGGACCAGCAGCGTCTCATCTTCGCTGGAAAAC
AGCTTGAGGATGGTCGTACTTTGGCCGACTACAACATCCAGAAGGAGTCTACCCTTCACTTGGTGCTA
AGGCTCCGTGGTGGTTTCTAAAAACTTATCTCTGTTATGAATCAGAAGAAGTTCATGTCTCGTTTCATTT
AAAACTTTGGTGGTTTGTGTTTTGGGGCCTTGTAAAGCCCCTGATGAATAATTGTTCAACTATGTTTCC
GTTCCTGTGTTATACCTTTCTTTCTAATGAGTAATGACATCAAACTTCTTCTGTATTGGAATTATGTCCTT
GTGAGTCTCTTTATCATCGTTTCGTCTT
>Unigene17976_All
CATTGAGTCGGATAGGGTCTTCTATATAAGGACGTCCTCTCACGCTTCTCTTCCTTCATCAGTCTCTGGT
TGAAAATAGCTCTAGATTCCAATTGATTTCAGCTTTTCTCTCCAATATCTCTTTTTCTCTCTTTAGTTTTG
GAAGATGCAGATCTTCGTGAAAACCCTAACCGGCAAGACCATCACCCTAGAGGTGGAGAGCTCCGAC

ACCATCGATAATGTAAAGGCCAAGATCCAAGACAAGGAAGGGATCCCTCCGGATCAGCAGCGACTCAT
CTTCGCCGGAAAGCAGTTGGAGGACGGCCGTACCCTTGCCGACTACAACATCCAGAAGGAGTCGACT
CTCCACCTTGTGCTCCGTCTCCGCGGCGGTATGCAGATCTTCGTCAAAACCCTAACCGGGAAGACCAT
CACCCTCGAGGTCGAGAGCTCTGACACTATTGACAACGTCAAGGCTAAGATTCAAGACAAGGAAGGC
ATCCCTCCGGATCAGCAGCGCCTGATCTTCGCCGGGAAGCAGTTGGAGGACGGCCGTACCCTTGCCGA
CTACAACATCCAGAAGGAGTCGACTCTCCACCTGGTGCTCCGTCTCCGCGGTGGGATGCAGATCTTTG
TCAAAACCCTAACTGGGAAGACCATCACCCTAGAGGTGGAGAGCTCCGACACCATCGATAATGTAAAG
GCCAAGATCCAAGACAAGGAAGGGATCCCTCCGGATCAGCAGCGATTGATCTTCGCCGGGAAGCAAT
TGGAGGACGGCCGCACCCTCGCCGATTATAACATTCAGAAGGAGTCGACCTTGCATCTGGTGTTGAGA
TTGAGGGGAGGGATGCAGATCTTTGTGAAGACTCTGACAGGGAAGACCATCACGCTGGAGGTTGAGA
GCTCGGACACAATAGACAACGTGAAGGCCAAGATTCAGGATAAGGAAGGGATTCCTCCGGACCAGCA
GAGGCTCATCTTTGCCGGAAAGCAATTGGAGGATGGGCGTACTCTCGCTGATTACAACATTCAGAAGG
AGTCGACTCTCCACCTCGTGCTCCGCCTCCGTGGCGGCATGCAGATCTTCGTCAAAACCCTAACCGGG
AAGACCATAACCCTTGAGGTCGAGAGCTCCGACACCATC

(3) *TUA*

>gi|214003724|gb|FJ228477.1| Betula pendula alpha tubulin mRNA, complete cds
ATGAGGGAGTGCATCTCAATCCACATTGGTCAGGCTGGTATTCAGGTCGGCAATGCCTGCTGGGAACT
TTACTGCCTCGAGCACGGCATCCAGCCTGATGGCCAAATGCCAAGCGATAAGACTGTTGGCGGAGGTG
ACGACGCTTTCAACACCTTTTTCAGTGAAACTGGTGCTGGAAAGCACGTCCCTCGGGCGGTCTTCGTT
GACCTTGAGCCTACTGTCATTGATGAAGTGAGGACCGGAACTTACCGCCAGCTCTTCCACCCTGAACA
GCTGATCAGTGGCAAGGAAGATGCTGCCAACAACTTTGCCCGTGGTCATTATACCATTGGAAAGGAGA
TTGTTGATCTGTGTTTGGACCGTATCAGAAAGCTTGCTGATAACTGCACTGGCCTCCAAGGATTCCTTG
TATTTAATGCTGTTGGTGGAGGCACTGGTTCTGGGCTTGGCTCCCTTCTACTTGAGCGTTTGTCGGTCG
ACTACGGCAAGAAATCAAAGCTGGGTTTCACGGTATACCCTTCTCCACAGGTTTCCACATCTGTTGTTG
AGCCTTACAACAGTGTCTTGTCTACCCATTCCCTCTTGGAACACACTGATGTGGCTGTACTTTTGGACA
ATGAGGCCATCTATGACATCTGTAGGCGGTCCCTTGACATTGAGCGACCCACCTACACCAACCTCAAC
CGCCTTGTCTCTCAGGTGATTTCATCTTTGACTGCCTCTCTGAGATTTGATGGTGCCCTGAACGTGGAT
GTGACTGAATTTCAGACTAACTTGGTCCCCTACCCCAGAATCCACTTCATGCTTTCTTCTTATGCACCA
GTCATCTCTGCCGAGAAGGCATATCACGAACAACTCTCGGTAGCAGAAATCACCAACAGTGCATTCGA
GCCATCATCTATGATGGCCAAGTGTGATCCTCGCCATGGCAAGTACATGGCTTGCTGCCTGATGTACCG
TGGTGATGTTGTGCCTAAGGATGTCAATGCTGCTGTTGCTACTATCAAGACCAAGCGCACCATTCAGTT
TGTTGACTGGTGCCCCACCGGATTCAAGTGCGGTATTAACTACCAGCCTCCCACTGTTGTTCCAGGAG
GTGACCTTGCCAAGGTGCAGAGGGCTGTTTGCATGATCTCCAACTCTACCAGTGTGGCTGAGGTGTTC
TCCCGCATCGATCACAAGTTTGACCTTATGTATGCCAAGCGCGCCTTTGTGCATTGGTACGTGGGTGAG
GGTATGGAGGAAGGAGAATTCTCAGAGGCTCGTGAGGACCTTGCTGCCCTCGAGAAGGATTATGAGG
AAGTTGGTGCTGAGTCAGCTGAGGGCGAGGATGATGAAGGGGATGAGTACTAA
>CL4792.Contig2
ATCATCGGTCTGTATTGGGCCATTGTCCAAAACCAAACAGAACATTGCCTCGTCGTTCGCCGCATCTTT
TGATAATTAAACGGTGTCGTTGCTTTGGCCCCTGATGCGGGCAAAGCAATTCAAATCTCAAATGATCGA
CCGTGAAAAAGGGACAAAGCGCGGTTAAATTTAGGGCGGTTGAGTCTGGCCAATAAGGATTAACCAC
GTGCACCCGCGCGTAACCACGGTTCGCTAACCGCGGTTAAACTCATTCCGATTCTCTCTCCGTCGCAAA
TAAATCTCCAGGCGAGAAATCAGTTCAGAGACACAACATTGTCGCAAACGAGAGGCGTCTTGATAAA
CGCATTTCTCTCGCATTCTCTCTAGCTTCTCGTCGATTCCTTTTAGTGTTTTTCAGCGAAAATGAGAGAG

TGCATCTCGATCCACATCGGCCAGGCCGGCATTCAGGTCGGCAATGCGTGCTGGGAACTTTACTGCCT
CGAGCACGGCATTCAGCCTGATGGCCAGATGCCAAGTGACAAGACCGTTGGGGGAGGTGATGATGCT
TTCAACACCTTCTTCAGCGAAACTGGTGCAGGGAAGCATGTCCCCCGTGCAGTTTTTGTAGATCTAGA
GCCCACTGTCATTGATGAAGTTAGGACTGGAACATACCGGCAACTCTTTCACCCTGAACAGCTTATCA
GTGGCAAGGAAGATGCTGCTAACAATTTTGCTCGTGGCCACTACACCATTGGGAAAGAGATCGTCGAT
CTTTGTTTGGATCGCATCCGAAAGCTTGCTGACAACTGCACTGGCCTCCAAGGATTCCTTGTATTCAAT
GCCGTTGGTGGAGGCACTGGTTCTGGGCTTGGCTCCCTTCTCTTGGAGCGTTTGTCCGTCGACTACGG
AAAGAAATCAAAGCTGGGTTTCACTGTCTACCCTTCTCCACAGGTTTCCACCTCTGTTGTTGAGCCTTA
CAACAGTGTCCTGTCTACCCATTCGCTCCTGGAACACACTGATGTGGCTGTACTTTTGGACAATGAGG
CCATTTATGACATTTGTAGGCGCTCCCTTGACATTGAGCGACCCACCTACACCAACCTCAACCGCCTTG
TCTCTCAGGTGATTTCATCTTTGACTGCCTCTCTGAGATTTGATGGTGCCTTGAACGTGGATGTGACTG
AATTTCAGACAAACTTGGTCCCCTACCCCAGAATCCACTTCATGCTTTCTTCTTATGCACCAGTCATCTC
TGCTGAGAAGGCATATCACGAACAACTCTCAGTAGCTGAAATCACCAACAGTGCATTTGAGCCATCAT
CCATGATGGCCAAGTGTGATCCTCGCCATGGCAAGTACATGGCATGCTGTCTGATGTACAGAGGAGAT
GTAGTGCCCAAGGATGTGAACGCAGCTGTGGCCACCATAAAGACCAAGCGCACCATCCAATTTGTTGA
CTGGTGCCCTACTGGATTCAAGTGTGGTATCAACTACCAGCCACCAACTGTTGTTCCTGGTGGTGATCT
TGCCAAGGTTCAGAGGGCAGTGTGCATGATCTCAAACTCCACCAGTGTTGCCGAAGTGTTCTCACGCA
TTGACCACAAGTTTGACCTTATGTATGCAAAGAGGGCATTCGTTCACTGGTATGTGGGTGAGGGTATGG
AAGAAGGTGAATTCTCTGAGGCCCGTGAGGACCTTGCCGCCCTGGAGAAAGATTATGAGGAGGTTGG
TGCTGAGGCAGGTGATGACGAGGAAGGTGACGAGGGAGATGAGTACTGAGAATGAATGCTTGTTTAG
TTACTTGCTTTCTAATGCTTCAGTTCTGTGGTGATGACTTGTGTTTTTCCTTTCCAAACTGTTGGCTTCT
AGTTGCCCTGGATTCTATTTGCTATTTCACTCAGCGTGAAACAGTCATGTTCGTTGTTTGGTTTACTACT
GTAATGACATCAAT

(4) *EF1-α*

>gi|145322982|ref|NM_001035916.2| Arabidopsis thaliana elongation factor 1-alpha 1 mRNA, complete cds
ATTCCTTGCTTCATTTTTCGTCACCCTAGCCGCTTTACTCTCTTGCGATATCTCTGAGGTAATTCTGGAG
CAGCATAAACAATCAATTGATTCATTCTTCTTGATACCAAGTTGTGGAAACTCTAGGATTGTTTGCTGTA
TATCTCCAGAATTGCTGTTTTGATTGAATTTAGGTCGCTTAGCTCAGTTGATAGAGCACCACATTTTTTG
TGGTAGAAATCGATTTGTTTGACAGTCTCTAACCATGGGTAAAGAGAAGTTTCACATCAACATTGTGGT
CATTGGCCACGTCGATTCTGGAAAGTCGACCACCACTGGACACTTGATCTACAAGTTGGGTGGTATTG
ACAAGCGTGTCATTGAGAGGTTCGAGAAGGAGGCTGCTGAGATGAACAAGAGGTCCTTCAAGTACGC
ATGGGTTTTGGACAAACTTAAGGCTGAGCGTGAGCGTGGTATCACCATTGACATTGCTCTCTGGAAGT
TCGAGACCACCAAGTACTACTGCACTGTCATTGATGCTCCTGGCCATCGTGATTTCATCAAGAACATGA
TCACTGGTACCTCCCAGGCTGATTGTGCTGTCCTTATCATTGACTCCACCACTGGTGGTTTTGAGGCTG
GTATCTCCAAGGATGGTCAGACCCGTGAGCACGCTCTACTTGCTTTCACCCTTGGTGTCAAGCAGATG
ATCTGCTGTTGTAACAAGATGGATGCCACTACCCCCAAGTACTCCAAGGCCAGGTACGATGAAATCATC
AAGGAGGTGTCTTCCTACTTGAAGAAGGTTGGTTACAACCCCGACAAAATCCCATTTGTGCCCATCTC
TGGATTTGAGGGTGACAACATGATTGAGAGGTCCACCAACCTTGACTGGTACAAGGGACCAACTCTCC
TTGAGGCTCTTGACCAGATCAACGAGCCCAAGAGGCCGTCAGACAAGCCCCTTCGTCTCCCACTTCA
GGATGTCTACAAGATTGGTGGTATTGGAACGGTGCCAGTGGGACGTGTTGAGACTGGTATGATCAAGC
CTGGTATGGTTGTGACCTTTGCTCCCACAGGATTGACCACTGAGGTCAAGTCTGTTGAGATGCACCAC
GAGTCTCTTCTTGAGGCACTTCCAGGTGACAACGTTGGGTTCAATGTTAAGAATGTTGCTGTCAAGGA
TCTTAAGAGAGGGTACGTCGCATCCAACTCCAAGGATGACCCTGCCAAGGGTGCTGCTAACTTCACCT

CCCAGGTCATCATCATGAACCACCCTGGTCAGATTGGTAACGGTTACGCCCCAGTCCTGGATTGCCAC
ACCTCTCACATTGCAGTCAAGTTCTCTGAGATCTTGACCAAGATTGACAGGCGTTCTGGTAAGGAGAT
TGAGAAGGAGCCTAAATTCTTGAAGAATGGTGATGCTGGTATGGTGAAGATGACTCCAACCAAGCCCA
TGGTTGTGGAGACCTTCTCTGAGTACCCACCACTTGGACGTTTCGCTGTGAGGGACATGAGGCAGACT
GTTGCAGTCGGTGTTATCAAGAGTGTTGACAAGAAGGACCCAACTGGAGCCAAGGTTACCAAGGCTG
CAGTCAAGAAGGGTGCCAAGTGAACTCTGAACATCAAAACTCTTTCCGCTGATGAAATGAAGGACTAT
TTTAGTTTCTTTACTTTAGTAGTTTGGTATTTGGTTGGTTGTTTGTGTTACCGCTTCGTTTATTCTCCGTC
GGAGCTCAATTCTCGGAATTGGGTTCTTGATCGGAGGTGGCGGAGGTACTTGGCACTCGAGCTTCCTG
TTTATTTCTTTTGTTTTGTGTTATGATTTTGAACCTTTTTGGATATTCGGTTATATGATATTTGTGTCATTCC
TGCGGATATTATCTTATCTTATCAACTTAAGTCTGTTTTTCTTCTCTAAAATTCAACGATCAGCCTCTGAA
GTTTCTTACATGGTAAAATTGTAATCTAGAACTTTAAACAACATTGGAGAAACAAGTCTATATCAGTTT
>CL828.Contig4_All
CTCGATTCTCATATCCTCATCTCTCTCGCTATTCACAAAGAAGACGGCGAGAGAGTCTCTTCAGCTATTT
CTTATCATCCTTCTTCGTCTGCCGCAGGCTTTTTAGAAGTTCATAATGGGTAAGGAGAAGGTTCACATC
AACATTGTGGTCATTGGCCATGTCGACTCTGGAAAGTCGACCACCACTGGGCACTTGATCTACAAGCT
TGGGGGTATTGACAAGCGTGTCATTGAGAGGTTTGAGAAGGAGGCTGCTGAGATGAACAAGAGGTCA
TTCAAGTACGCCTGGGTGCTTGACAAGCTCAAGGCTGAGCGTGAGCGTGGTATCACCATCGATATTGC
CTTGTGGAAGTTCGAGACCACCAAGTACTACTGCACTGTCATCGATGCTCCCGGACATCGTGACTTTAT
TAAGAACATGATTACTGGGACTTCACAGGCCGACTGTGCTGTCCTTATCATTGATTCCACCACTGGTGG
TTTTGAAGCTGGAATCTCCAAGGACGGCCAGACCCGTGAGCATGCTCTCCTTGCCTTTACCCTTGGTG
TCAAGCAGATGATCTGTTGCTGCAACAAGATGGATGCAACTACCCCTAAATACTCCAAGGCGAGGTAT
GATGAAATTGTGAAGGAAGTTTCTTCCTACCTGAAGAAGGTTGGTTACAACCCTGACAAAATCCCATT
TGTTCCCATCTCTGGTTTCGAGGGTGACAACATGATTGAGAGGTCAACCAACCTTGACTGGTACAAGG
GCCCAACCCTCCTTGATGCTCTTGACCTGATCTCTGAACCCAAGAGGCCCTCAGACAAGCCTCTCCGT
CTCCCTCTTCAGGATGTTTACAAGATTGGTGGTATTGGAACTGTCCCAGTTGGTCGTGTTGAGACCGGT
GTCATCAAGCCTGGTATGGTTGTTACCTTTGGCCCAACTGGACTAACCACTGAAGTTAAGTCTGTTGAG
ATGCACCACGAGGCTCTTCTGGAGGCTTTGCCAGGTGACAATGTTGGCTTCAACGTCAAGAATGTTGC
TGTGAAGGATCTCAAGCGTGGTTTTGTTGCATCCAACTCAAAGGATGACCCTGCCAGGGAAGCTGCTA
ACTTCACATCTCAGGTCATTATCATGAACCACCCTGGTCAGATTGGAAATGGTTATGCCCCAGTTCTCG
ACTGCCACACCTGCCACATTGCTGTCAAGTTTGCTGAACTTGTCACCAAGATTGACAGGCGTTCTGGT
AAGGAGCTCGAGAAGGAGCCCAAGTTTTTGAAGAACGGTGATGCAGGGATTATCAAGATGATTCCCA
CCAAGCCCATGGTTGTTGAGACTTTCTCTGAGTACCCACCTCTTGGTCGCTTTGCTGTCAGGGATATGC
GCCAGACTGTTGCTGTTGGTGTTATCAAGGGTGTTGAGAAGAAGGATCCCTCTGGTGCTAAAGTCACC
AAATCTGCTGCCAAGAAGAAGTGAATGGTGCGGTCACCCATTTTCATTGATGCTGAGAACAATTTATAT
CTCTATTACTGTGTGCTTTTTCAATTTCTAGACGGTTTTGTCTCTAGTTTTATTTCTATTGGCTTTACGTGT
TCCTCCTTGTCTGGTCGTCGCACCCAGAACTGGGTGCTAGACAGGCGGTGGTAACGCATTGCCAGTGT
TCGAGTCCTGGAGTTTTTAACACCCTTTTATTGTGGATGTTGAGTTAATTTAAATTTAAGGTTTTTAATTT
AGTATTAGTCACCTGTTTGCTGTCTCCGTTTAAATTGCTTTAGGTTTTTAGTTTGTTTCTTCGTGTGGATT
CAATTTTGGGCAGCCCTCAATGGCTTTTGCCTGTTGAACTAGGGACTTGTTTTCCCTATACGGTGGTAA
GTGGTATGTGTTGGATGATGCATATATTTCAAAAGCTTCATCAATCTTGGTAAGGTTTGTGTTGGCTACT
GATAATGTAGTCATTTCTTTGCACTATAGTTGTGAAGCTAAAACGTTTAGGGCATTGAGCAAATTAT

(5) *GAPDH*

>gi|283480608|emb|FN396887.1| Populus trichocarpa partial gapdh gene for glyceraldehyde-3-phosphate, exons

8-11
CTTAGCTTCATGTTATATCCTGATTTATCTTGTTGCTTTCTTTGCATTTGGGTTTGAAAGTTAGTCCTCTG
ATGCTCTGTATCCATGTTATTCAGCCACTCAGAAAACTGTTGATGGTCCATCAATGAAGGACTGGAGAG
GTGGAAGGGCTGCTTCCTTCAATATCATTCCTAGCAGCACTGGGGCTGCCAAGGTATGCATTTTTTTGG
GGGGAGCATCATTGTTTGTTTATAGTTTATTGCTATGTTTATGCACCTACGGCCGGACCTTATTCTTCTTC
GTTCATTGTCAAATCTCATAGGCTGTTGGAAAGGTTCTGCCAGCACTTAATGGAAAATTGACTGGAATG
TCCTTCCGTGTTCCTACTGTGGATGTCTCTGTTGTTGACCTCACTGTCAGGCTTGAGAAGAAGGCAACA
TACGAGGCTATCAAATCTGCTATCAAGTAAGTGCTTGTTAGAATGATCTTCTAGTCAGAATCTTCTAGCT
TATTTATATTCTGATTTTATTTGTTTACCTTGATGATCTCCAGGGAGGAGTCTGAGAATAACCTCAAGGG
TATTCTGGGTTACGTTGAAGAGGATGTGGTGTCTACTGACTTCATTGGTGACAGCAGGTAAAGGAAAA
GGCTGTGATGCTGTCTTCGTAGACCATGTGGATTGTGCTGGCCTGAATACATGCTTTCTTTGTATTTCCT
CTAATGGTTGTGCTTTATGCAGGTCAAGCATATT
>Unigene22682_All
CGGGCGTTCAATCTCTGTCTCTCTTACGTTCTCAGTTCCAATCTATACGCTCATTCCGGCGCTATCTCCT
CCAATCATCATCATGGCCTCGCACAAGAAGATCAAGATCGGAATCAACGGGTTCGGAAGGATTGGTCG
TTTGGTTGCCAGGGTTATTCTTCAGAGAAACGATGTTGAGCTCGTCGCTGTTAACGATCCCTTCATCAC
CACTGACTACATGACATATATGTTCAAATACGACACCGTTCACGGGCACTGGAAGCACACTGACGTGA
AGGTCAAGGACTCCAAAACCCTTCTCTTCGGCGAGAAGCCCGTCTCAGTTTTCGGCTTCAGGAACCC
AGAAGAGATCCCATGGGCCCAGGCTGGAGCCGATTACGTCGTCGAGTCTACTGGGGTTTTCACCGACA
AAGAAAAGGCTGCTGCCCACTTGAAGGGTGGTGCTAAGAAGGTGATCATTTCTGCCCCCAGCAAAGA
TGCACCCATGTTTGTTGTGGGTGTGAACGAAAAGGAGTACAAGCCCGACCTTGACATTGTTTCCAATG
CTAGCTGCACTACCAACTGCCTTGCTCCTCTTGCTAAGGTTATTAATGACAGGTTTGGAATTGTTGAGG
GTCTCATGACCACCGTCCACTCCATCACCGCCACACAGAAGACTGTTGATGGCCCCTCAAGCAAGGAC
TGGAGGGGTGGAAGAGCTGCTTCCTTCAACATTATTCCCAGCAGCACCGGAGCTGCAAAGGCTGTTG
GAAAAGTTCTTCCGTCACTTAATGGCAAATTGACTGGAATGTCTTTCCGTGTACCCACTGTGGATGTTT
CAGTTGTTGACCTCACTGTCAGGCTTGAGAAGAAGGCAACCTATCAGGAGATAAAAGATGCTATTAAG
GAGGAGTCTGAGGGCAAGCTCAAGGGCATTTTGGGTTACACCGAGGATGATGTGGTGTCTACCGACTT
TATTGGTGACAGCAGGTCTAGCATTTTTGACGCAAAGGCTGGAATTGCATTGAATGAGGGCTTTGTGA
AACTTGTCTCTTGGTATGACAATGAGTGGGGCTACAGTACTCGTGTGGTTGACTTGATTGCCCACATCG
CATCTGTATGTTGAAGCTAAACGGAATATCCAAGGCTGCTTTCGATGTGTTTTTTGCGTCTACGGTTTAG
TTATATGGCTGCACTCCATTAGGAGTTTGGAATAAACTTGAGTCTTATTGGTATCTTTCTGTTTAACTGCT
GATCTAGGTGGCTTTTGGTCCCCGTTTGATATTTTGTTTGGGAGTTGTAGGGAATTTCCTTTCTTTTTTG
TTAAAAGTAAATGCATATTGGAACTTTATTTCTTGAGATTTTGAAACGAAGCATAAGAACATATTGAATG
TGGAATTCAAGCAAGCTTGATTGAGGCTCTAGCTGCTAACCCATATTTCATTCAATTAGGTCACACGGC
CTTGCTCTCATGAACCGTGGGTTAAGTGTCTGGCTCCATTTCTCA

(6)18*S rRNA*
>Unigene3338_All
AATCCAAGAATTTCACCTCTGACAGCTGAATACTGACGCCCCCGACTATCCCTATTAATCATTACGGCG
GTCCTAGAAACCAACAAAATAGGACCGCACGTCCTATTCTATTATTCCATGCTAACGTATTCGAGCAAA
GGCCTGCTTTGAACACTCTAATTTTTTCAAAGTAAAAGTCCTGGTTCCCCAACACACCCAGTGAAGGG
CATGAGGTTCTCCAGAAGGAAAGGCCCGGCCGGACAAGTACACGCGGTGAGGCGGACCTGCCAGCC
AGGCCCAAGTTTCAACTACGAGCTTTTTAACTGCAA
>gi|51235019|gb|AY652861.1| Populus trichocarpa 18S ribosomal RNA gene, partial sequence

TGTCTCAAAGATTAAGCCATGCATGTGTAAGTATGAACTAATTCAGACTGTGAAACTGCGAATGGCTCA
TTAAATCAGTTATAGTTTGTTTGATGGTATTTGCTACTCGGATAACCGTAGTAATTCTAGAGCTAATACGT
GCAACAAACCCCGACTTCTGGAAGGGACGCATTTATTAGATAAAAGGTCGACGCGGGCTCTGCCCGTT
GCTCTGATGATTCATGATAACTCGACGGATCGCACGGCCTTCGTGCTGGCGACGCATCATTCAAATTTC
TGCCCTATCAACTTTCGATGGTAGGATAGAGGCCTACCATGGTGGTGACGGGTGACGGAGAATTAGGG
TTCGATTCCGGAGAGGGAGCCTGAGAAACGGCTACCACATCCAAGGAAGGCAGCAGGCGCGCAAATT
ACCCAATCCTGACACGGGGAGGTAGTGACAATAAATAACAATACCGGGCTCTTCGAGTCTGGTAATTG
GAATGAGTACAATCTAAATCCCTTAACGAGGATCCATTGGAGGGCAAGTCTGGTGCCAGCAGCCGCGG
TAATTCCAGCTCCAATAGCGTATATTTAAGTTGTTGCAGTTAAAAAGCTCGTAGTTGGACTTTGGGTTG
GGTCGGCCGGTCCGCCTCAGGTGTGCACCGGTCGCCTCGTCCCTTCTACCGGCGATGCGCTCCTGGCC
TTAACTGGCCGGGTCGTGCCTCCGGTGCTGTTACTTTGAAGAAATTAGAGTGCTCAAAGCAAGCCTAC
GCTCTGGATACATTAGC
ATGGGATAACATCATAGGATTTCGATCCTATTGTGTTGGCCTTCGGGATCGGAGTAATGATTAACAGGG
ACAGTCGGGGGCATTCGTATTTCATAGTCAGAGGTGAAATTCTTGGATTTATGAAAGACGAACAACTG
CGAAAGCATTTGCCAAGGATGTTTTCATTAATCAAGAACGAAAGTTGGGGGCTCGAAGACGATCAGAT
ACCGTCCTAGTCTCAACCATAAACGATGCCGACCAGGGATTGGCGGATGTTGCTTTTAGGACTCCGCC
AGCACCTTATGAGAAATCAAAGTTTTTGGGTTCTGGGGGGAGTATGGTCGCAAGGCTGAAACTTAAAG
GAATTGACGGAAGGGCACCACCAGGAGTGGAGCCTGCGGCTTAATTTGACTCAACACGGGGAAACTT
ACCAGGTCCAGACATAGTAAGGATTGACAGACTGAGAGCTCTTTCTTGATTCTATGGGTGGTGGTGCA
TGGCCGTTCTTAGTTGGTGGAGCGATTTGTCTGGTTAATTCCGTTAACGAACGAGACCTCAGCCTGCTA
ACTAGCTATGCGGAGGTGACCCTCCGCGGCCAGCTTCTTAGAGGGACTATGGCCTTCCAGGCCAAGGA
AGTTTGAGGCAATAACAGGTCTGTGATGCCCTTAGATGTTCTGGGCCGCACGCGCGCTACACTGATGTA
TTCAACGAGTCTATAGCCTTGGCCGACAGGCCCGGGTAATCTTTGAAATTTCATCGTGATGGGGATAGA
TCATTGCAATTGTTGGTCTTCAACGAGGAATTCCTAGTAAGCGCGAGTCATCAGCTCGCGTTGACTACG
TCCCTGCCCTTTGTACACACCGCCCGTCGCTCCTACCGATTGAATGGTCCGGTGAAGTGTTCGGATCGC
GGCGACGTGGGCGGTTCGCCGCCGGCGACGTCGCGAGAAGTCCACTG

(7)*TUB*

>gi|460240128|gb|KC243679.1| Salix arbutifolia beta-tubulin 6 mRNA, complete cds
ATGCGTGAAATTCTTCACATCCAAGGAGGCCAATGTGGCAATCAAATCGGTGCAAAGTTCTGGGAGGT
GGTATGCGCAGAACATGGGATTGATGCAACAGGAAAATACAACGGAGATTCTGAGCTGCAGCTTGAA
AGGATCAATGTTTATTACAATGAGGCAAGTGGTGGCAGGTTTGTCCCGAGGGCTGTCCTAATGGACCT
TGAACCTGGTACCATGGACAGTGTCAGGTCAGGTCCTTATGGTCAGATTTTCAGACCTGATAATTTTGT
TTTCGGACAATCTGGTGCTGGAAACAACTGGGCTAAAGGCCATTACACCGAGGGTGCTGAGTTGATTG
ATTCTGTCCTCGATGTTGTCCGAAAAGAAGCCGAGAATTGTGATTGCTTACAGGGATTTCAGGTGTGCC
ATTCATTAGGAGGAGGGACCGGATCTGGTATGGGCACTCTATTGATATCGAAGATCAGAGAGGAATACC
CGGATCGGATGATGCTTACGTTCTCTGTCTTTCCATCCCCTAAGGTTTCTGATACAGTTGTTGAGCCTTA
TAATGCAACCCTTTCTGTGCATCAGCTTGTTGAAAATGCAGATGAATGCATGGTCCTTGATAACGAAGC
TCTCCACAACATCTGCTCCCGCACCCTCAAACTCACCTCGCCAAGCTTTGGTGATCTAAATCATTTGAT
CTCTGCTACCATGTCCGGGGTCACGTGTTGCTTGAGATTTCCAGGACAGCTTAACTCTGACCTGAGAA
AGCTTGCTGTGAATCTGATCCCTTTCCCTCGGCTACATTTCTTCATGGTGGGTTTCGCTCCTCTGACCTC
ACGTGGTTCACAGCAGTACAGCACCTTGTCCGTCCCAGAGCTCACCCAGCAAATGTGGGATGCCAAG
AACATGATGTGCGCAGCAGACCCAAGCCACGGTCGGTATCTGACAGCCTCGGCCATGTTCAGGGGCA
AGATGAGTACCAAGGAAGTGGATGAGCAGATGATGAATGTTCAAAACAAACACTCATCGTACTTTGTT

GAATGGATCCCAAACAATGTAAAATCAACTGTTTGTGATAGTCCTCCCACAGGCCTAAAAATGGCATC
GACATTCATCGGAAACTCCACATCAATTCAGGAAATGTTCCGTCGCGTGGGCGAGCAGTTTACTGCCA
TGTTTAGGAGGAAGGCTTTCTTGCATTGGTACACTGGGGAAGGAATGGATGAGATGGAGTTCACAGAG
GCAGAGAGCAACATGAATGATTTGGTGTCCGAGTACCAGCAGTACCAAGATGCCACAGCTTGCGAGG
ACTATGAGGATGAAGAGGAGGAACTCCATGACATGTGA
>CL9540.Contig5_All
CCTCTCTCTCTCTCTCTCTCTTTATTGTATCTCTATTTAAAATCTTTCATCCCCGTTATCTTTACTCTCCATA
TTGTCGCTTTATTTTCACTTTACTCTCTCTCTCTTTCTCTCTGCAAACCCTAACGCTCTGCAAAAATGCG
AGAAATCCTTCACATCCAAGGAGGCCAGTGTGGCAACCAAATCGGTGCCAAGTTCTGGGAAGTGGTC
TGTGCCGAGCACGGCATCGATTCCACGGGACGGTACCAGGGGGATTCTGAGCTTCAATTGGAGAGGG
TCAACGTGTATTACAATGAAGCCAGTTGTGGCAGATTTGTACCCCGTGCCGTTCTCATGGATCTGGAGC
CCGGGACCATGGACAGCGTGAGGTCCGGTCCGTATGGTCAGATTTTCAGGCCAGACAACTTCGTTTTC
GGCCAGTCTGGTGCTGGAAACAACTGGGCCAAAGGGCATTACACTGAGGGTGCTGAGTTAATCGATT
CCGTCCTTGATGTTGTTCGAAAAGAGGCCGAAAATTGTGACTGCTTGCAGGGGTTCCAGGTGTGCCAC
TCTTTGGGTGGTGGAACTGGATCTGGAATGGGGACGCTTCTGATTTCGAAGATTAGGGAGGAGTACCC
AGATAGGATGATGATGACTTTTTCGGTCTTTCCGTCGCCTAAGGTCTCCGACACTGTTGTGGAGCCTTA
CAATGCCACTTTGTCTGTTCATCAGCTTGTTGAAAATGCAGATGAGTGCATGGTGCTTGACAATGAGGC
CCTCTATGACATTTGCTTTCGAACGCTGAAGCTCACCACTCCCAGCTTTGGCGATTTGAACCATTTAATA
TCTGCAACCATGTCTGGAGTTACATGCTGCCTTAGGTTCCCAGGCCAACTCAACTCAGATCTACGGAA
ACTTGCTGTGAATCTCATTCCATTTCCTCGTCTCCACTTTTTCATGGTCGGGTTTGCACCCCTGACTTCA
CGAGGATCTCAGCAGTACCGTGCTCTGACAGTTCCTGAGCTCACCCAACAAATGTGGGATTCAAAGAA
CATGATGTGTGCAGCTGACCCGCGACATGGCCGTTACCTGACAGCATCCGCTATGTTCCGTGGGAAGA
TGAGCACCAAGGAAGTAGATGAACAGATGCTGAATGTGCAGAACAAGAACTCCTCATACTTTGTTGA
GTGGATTCCCAACAATGTGAAGTCAACAGTTTGTGATATCCCACCTACTGGTCTCAAGATGGCATCAAC
CTTCATTGGTAATTCGACATCCATACAGGAGATGTTCCGAAGGGTGAGTGAGCAGTTTACTGCAATGTT
CAGGAGGAAGGCTTTCTTGCATTGGTATACTGGGGAGGGAATGGATGAAATGGAGTTTACCGAGGCGG
AGAGCAACATGAATGATCTGGTTGCAGAGTATCAGCAGTATCAAGATGCAACCGCTGATGAGGAGGTA
GATTATGAAGACGAGGAGGAGGAGGCGGAGTATCAGGACTGATGATGGTGGATGGGTTATTGGGGAA
CTTGCTAGCTGATAGTAGCAAGTCTTTGTTTTTTATAACCTCAACTTCCAGTTGCTGGTTTGTAGCATTT
GACTAAGGAAAGACAATTATATTCTGCTGTGTAACTTTCATAATTTTCGAGCTTTTCTTAAATTCTCTTTC
TTGCTTTCTAGTTTTATGTATGAGAAGTGCTACGCTACACATCTTCTTATTCTTTTCTTTTTGCTCTTTAA
AATCATCATTACATCTGTTAATTCATATAGCTCCAACTAATAGTGATTTTAAGACGTGGGAAGATAAGAG
AAGGATCGGGAGAAAATGAAGCATTTTCTATTATGTATGCATATA

Three new candidate reference genes screened from transcriptome data

(1) *ChaSTP5*

>Unigene27057_All
ACCCCAACCCAACCACGGTTAAGCCCCACTCCCGTAGCAGTTTATAAACGTGCCCACCTTGCCGGTGA
TTCAGTAAACAATCAATCTCAAACCCCCTCTCCAAAGATGATGCTCGGAGAGCAACATCGTCCGAATC
CCACGGTCCACGTCCCTCCGTGGACACTCCAAGATGATCCAGCGGCCGATTTGTACTCCTCTTACGCTT
TAACCCCCCTCTCCGCGAACTCCGACGGCAATGGCAACGGCAGCGGGGAGTACTCCCCTTATTATCTC
CAGGAAGCCCTAACCGCGCTCCAGCGTTACTTGCCCTCCAACGGAAGCCCCGAGATGGACTCGGACT
CCGAAATAACGGGGTCGGACGCGCCGGTCGACGCGTACTCTTGCGACCATTTCCGGATGTTTGAGTTC

AAGGTGAGGCGGTGCGCACGTGGAAGGTCACATGACTGGACCGAGTGTCCGTACGCCCACCCAGGTG
AGAAGGCCCGCCGCAGAGACCCGAGAAAGTATCACTATTCCGGTACGGCGTGCCCCGATTTCCGAAA
GGGCAACTGTAAGAAGGGCGACTCTTGCGAGTTTGCGCATGGCGTGTTCGAGTGCTGGCTCCACCCG
GCTCGCTATCGCACGCAGCCATGCAAGGACGGACGAGCTGTCGCCGGAGGGTCTGTTTCTTTGCTCA
CACGACGGAGCAGCTCCGGGTTTTGCCTCAGCAGAGCCCGAGAAGCGTCGGAAGCTCGGTGAACTC
AACCGAGTCGTACGACGGGTCGCCGATGAGGCAGGCGTTTGAGGCCTCCTGTGTCAAAACACTGCCT
TTTCTGTCCACCCCATCGTCTGTTTCCCCGCCGGCGACCCCTCCGGCGGAGTCTCCGCCTATGTCGCCG
ATGACCCAGTCGCTGAGTCGTTCACTGGGTTCAAGTGCGATCAATGAAATGGTGGTCTCGCTTCGTAAT
TTACAGCTCGGAAAGGTCAAGTCTTTGCCATCCTCCTGGAACGTTCCAATCGGGTCTCCCGTGTTTGG
GTCCCCGAGAGGCTCCATCCTCCGACCCGGTTTCTGCAGCGTGCCTACGACCCCGACCCGGGCCGCCC
CTGCCCGTCCTGGAATCGGGTACCATGATATCTGGGACCAGTGTTGCGAGGAGGAACCGGCGATGGAG
AGGGTCGAGTCCGGGCGGGATCTGCGTACTAAGATGTTTGAGAAGCTGAGCAAAGAGAACTCCTTGG
GTCGGGCCGACCCGAACCCGTCGACCGGTGCTCCGGATGTGGGTTGGGTCTCAGAGCTGGTGAAGTG
AGCAGCAGCCCAATGAATCGGGACACGTGGGTGGTTGAGATAATCCCCGATGATTTCTTTTGCTAAAG
ATCTTTGATTCTATTTTGGTGACTTTATTTTGTTCCTTCTCTCACACTGTGAATAGTGTCTGAAGCAAAG
TGAGAGGACGGCTCTAGAATTTGATTTTGGAAGCAACAACTCGAAGATTCTGTGTATATATTTAATATTA
TCAGATGGTATGGGGGTGAAGAGTAGAATCTCTTTTTTGGGGGCCTGAGAACTCGGCAGAAGACATTA
GAAGATTGTTAAGCAAGCAAGCAACGAAAACACAAAAAAAAAAAAAAGAACTTTTTTTTGTATAGGTC
GCAGTGGATCATTCCCTTTTTCTTTCTGTTAATTATTGCTGTGTTTCTTCTCATTATCACCATCCTACTTAC
TGATCCTGAATCTTGATCCTTGTAAATATTATTTCTTAGCATGTATTTGGTCTTATCCTAATGCAATTATTA
ATGTATTGTATATTTTTCATCATTGAATTTGGG

(2) *ChaTF*

>Unigene8224_All
GAAGAGCACGACAGTAGATCTGGCAGCGATAACATGGACGGCGGTTCTGGGGACGACCAGGACGCA
GCCGACAACCCTCCGAGAAAGAAGCGTTACCACCGACATACACCACAGCAAATCCAAGAACTCGAAG
CTCTGTTCAAGGAGTGCCCTCATCCTGATGAGAAACAAAGAATGGAGCTTAGCAAGCGGCTTTGCTTA
GAAACCAGACAGGTGAAATTCTGGTTCCAAAATCGCCGTACCCAAATGAAGACCCAATTGGAGCGCC
ACGAGAATTCATTGCTCAGGCAAGAGAACGATAAGCTTAGGGCCGAAAATATGTCTATTAGGGATGCG
ATGAGGAGCCCCATTTGCTCCAACTGCGGCGGCCCAGCAATTATTGGAGAAATTTCACTCGAAGAGCA
GCATCTTAGGATTGAGAATGCCCGGTTAAAGGATGAGTTGGACCGTGTTTGCGCGCTCGCCGGAAAGT
TTCTGGGCCGTCCGATTTCATCTTTGGCTGCGTCAATCGGTCCTCCACTGCCAAGCTCAAGTTTGGAA
CTCGGCGTGGGGAGCAATGGTTTTGCTGGCTTGAACGCCGTGCCTACAACATTGCCTGATTTCGGTGT
TGGGAATATTTCCAGTGCTTTGTCGGTGGTTCCTCCGCCGGGTAGAGCAGGGGGCGGCGTGACGGCTC
TTGATCAGAGGTCAATGTTTCTAGAGCTTGCTGTGGCTGCCATGGATGAATTGGTTAAAATGGCGCAGA
CTGATGAGCCCCTCTGGCTCAGGAGCTTGGAAGGTGGGAGAGAAATGTTGAACCTCGAGGATTATATG
AGAAGCTTCACTCCTTGCATTGGCTTGAAACCCAACGGTTTTGTAACCGACGCTTCCAGGGAGACTGG
TATGGTCATTATCAACAGCTTGGCTCTTGTTGAGACGCTAATGGACTCGAATCGATGGGCGGAGATGTT
TCCTTGTATGATTGCTAGAGCGTCGACCATTGATGTGATATCTAGCGGCATGGGGGGAACTAGAAATGG
CGCACTTCAACTGATGCATGCTGAGCTGCAAGTCCTCTCGCCTTTGGTTCCGGTTCGGGAGGTGAATT
TCCTCCGATTTTGTAAGCAGCACGCGGAGGGCGTGTGGGCTGTGGTCGATGTATCCATTGACAGCGTC
CGAGAAACTTCGGGTGCACCAACATATGCCAACTGCAGGAGGCTTCCTTCTGGGTGCGTTGTGCAAGA
TATGCCTAATGGCTACTCCAAGGTTACATGGGTTGAGCATGCAGAGTACGATGAGTGCCAAGTCCACC
AGCTCTACCGGCCGTTGTTAAGCTCCGGCATGGGTTTCGGTGCCCAACGCTGGATCGCCACCCTTCAA

CGCCAATGTGAGTGCCTAGCCATCCTCATGTCCTCCGCCGTCCCCAGCCGAGATCACACCGCAATAACT
GCCAGTGGGCGACGAAGCATGTTGAAGCTGGCGCAACGAATGACCGACAACTTTTGCGCCGGGGTTT
GCGCGTCCACTGTGCACAAATGGAACAAGCTCAACGCTGGGAATGTGGACGAGGATGTCAGGGTGAT
GACCCGGAAGAGC

(3) *ChaUBC*

>Unigene376_All

GAAAGAGTGAATCTTTAATCCTCTCTAAAACATCTGAAGTGTCAACATCAAAGTATGGCTTGTAAGCA
GCAACCGTGAATGTCCGAAACCAACCACTCTGCTGGGGTTCATCAGAACCAGATACAGGTTTTGAAAA
TGTATCGTCGGCATCTCGAGGAGGGCGAGAAACACCTGAGATCTTCCCTTGCGCTTCGGGTGGAGGAA
ACGTCTGAAGAGTGGAATCGGCGAAGTTGACGGTGAGGTGGCCTGGATCTGGCACAGAGGCTACAGA
TCCTGAGACCTTCTGGTTGTCTATGCTTGTGTACTTGCCTGACATCATCTTTTCTCTCCCTTGTCTGCCT
CCTCTCCCTCTCCCTCTCCCTCTCGAACGGTCACGGAATTGGAGCTCGTCCAAAGCGAAAATAGTGAA
AGTTTGCTATTATTGGCATCCCATTTAAGCTCCGATTCCTCTTCGTTTTCTCCTGTTCGGTAAGAAGTCC
CTCTCTCTGTCTCCTCGTGTTCTTGGTCTTCTCCTTTGTAATCTCATACACTAATGATTTCACAGATCTCG
AGTTGATTGATTTTATTAATTACACTTCTCGGGATTCGTGGTTTCACTGTCCCGTGTGTTTGTCTGTCTCT
GCGAAATGTCTTAGCCCTGGTTGATTTCTAGGTTGTCCATTCTTAGGTCTAGGGCTTCAATTTGTTCCTT
TCTTTTTTGGTTGTTGTATGTAAATTCGAAATCAAATATAGGGGCTGTTTTGATCATCTGGGGATGAACT
AGGTGATCTTTGTAATTATGAAAATGGCCGAATTCTTGTGAATTGTAAATATCTTTCGGTTAGGTTATTGT
ACATTTGATTCAAAGCTTTTCTGACTTGTGTAGTAGTAACCAAACAATAAAATGTCGTCCGCCGGTGTC
ACTATTCTTAATCCGGTTCTCAATGAGCCTGTGGTTTTCCCAACTCATTTGTATTCAAAATTGGATCTGT
GCTCGAAAGAATCGATTTTGATATATCTCTCCATTTCGGGTTCAATGACTCCCATGCGTGTTTTGGAATC
TGATTCCATCGAGTCCATGAAGCTCAGAATTCAAACCTGTAAAGGGTTTGTTGTTAAGAACCAGAAGC
TGGTTTGCGGTGGCCGGGAATTGGCTCGGAGCAATTCTCTGCTCCAGGACTATGATGTTGCGGATGGG
AATGTTCTGCATTTGGTTCTTAGGCTTTCGGATCTTCAGGTAATTACTGTTAGGACTGTGAGTGGGAAA
GAATTTACATTCCATGTGGAACGAGATAGAGATGTTGGGTATGTGAAACAAAAGATTGTGAAGGAGGG
GAAGGAATTTGTTGATTTTGAAGAGCAGGAAGTTGTGTTGAATGGAGAGCGACTTGAGGATCAGAGG
CTGATCGATGATATCTGTAAACATAATGATGCAGTGATACATTTGTTGGTTCAGAAATCTGCAAAAGTCC
GTGCTAGACCAATTGAGAAGAATTTTGAGTTGTCTATTGTCGCACCACAGTTGAATGATGGGAGAGATT
ATGACGGTGATGGAGAAAAGTGTAGCAGACAATATGATGTTGGAGAAATAGTACCGAGGAAACCTCCT
GACAGTGATTTTTTGTTGGAGCCAGTTATTGTTAACCCCCAGATTCAATTGCCCTCTGTGATATGGGATA
TGGTTAACTCTACATTTGACGGATTAGATAGTGGTCATTACCCGATCAGGTCTATGGAGGGTACAGGAG
GAGTTTATTTTATGATGGATTCTTTGGGGCAGAAGTATGTGTCTGTTTTTAAGCCCATGGATGAGGAGCC
AATGGCTGTGAACAACCCCCAAGAGCTACCATATTCCCTGGATGGTGAGGGATTAAAAAAGGG
CACGAGAGTCGGAGAAGGAGCATTCAGGGAAGTTGCAGCTTACATTTTGGATCATCCGAAGGGTGGG
TACTACCCATTGTTTGACGATGAGAAGGGTTTTGCTGGGGTTCCCCCTACTTTTATGGTCAAGTGCTTG
CATGGAGGATTCAACCATCCAGGGGATTTGACTGTCAAGATTGGGTCTTTGCAGATGTTCATGGAAAAT
AACGGAAGTTGTGAGGATATAGGCCCTGGGGCTTTTCCTGTTCAGGAAGTGCATAAGATTTCTGTTTTG
GATATAAGGCTAGCAAATGCGGATAGGCATGCAGGCAATATTTTGCTGAGCAAAGCTGGAGAAGATGG
CCAGCCTATACTTATTCCGATTGATCATGGGTATTGCTTGCCTGAAACTTTCGAAGATTGCACATTTGAA
TGGCTCTATTGGCCACAGGCTCGCCAATCTTACTCCACAGAGACGATTGAGTACATAAGATCGCTGGA
TGCTGAAAAAGATATTGACCTTCTGAAGTTCCATGGATGGGAGTTGCCACTTGAATGTGCTCGCACGC
TCCGCATCTCAACTATGCTTCTGAAAAAGGGGGTGGAGAGAGGGCTCACCCCCTTTGCCATTGGAAGC
ATAATGTGCAGAGAAACCTTGAAGAAGGAATCTGTGATTGAGGAGATTGTCCAAGAAGCACAGGATT

CTGTGCTTCCTGGCACCAGTGAAGCTACATTCCTTGATGCCGTGTACCAAATCATGGATCGCTACCTTG
ACGTGATTGCTGGATCGCCTTTATCATAAGGATGCTGGTTTTTCTTTTCGGTTTTTGGAATACAAGTATAT
ATAGGTATGGCAAGGCAACTAGGATTTTCTCCTTTTGTCCGTCCACACACAATGTGATTGGAGTAACAT
TTCTTTGTGATTATCCATTCTTTCATATCAAATTATCAATCACTAGTGTGTTGTGATTGAACTGAAGTCAA
GTTTTTTAAGTTACAGATTCACTTCTTTAAGTTTTTTCTTTTATTATTTTTTTGAACGAGGGCCTCTCAGC
TTGAAGGGGTAGTGTTGTGTGATTCAATACAACGGATTGTTTGACATGGTAGAAGTGATGAAGCTTAAA
TCCATTGAGGTTTTGTAGTCCACCAAAACATCAGATTTTTTAACGTGGCAGAAATGATAAACCCTAAAT
TGATTGAGGTTTTGTTGTCCG
